# Extracellular matrix modulating enzyme functionalized biomimetic Au nanoplatform-mediated enhanced tumor penetration and synergistic antitumor therapy for pancreatic cancer

**DOI:** 10.1186/s12951-022-01738-6

**Published:** 2022-12-10

**Authors:** Xiao-Yan Yang, Jin-Guo Zhang, Qiao-Mei Zhou, Jie-Ni Yu, Yuan-Fei Lu, Xiao-Jie Wang, Jia-Ping Zhou, Xin-Fa Ding, Yong-Zhong Du, Ri-Sheng Yu

**Affiliations:** 1 Department of Radiology, Second Affiliated Hospital, School of Medicine, Zhejiang University, 88 Jiefang Road, Hangzhou, 310009 Zhejiang People’s Republic of China; 2 Institute of Pharmaceutics, College of Pharmaceutical Sciences, Zhejiang University, 866 Yuhangtang Road, Hangzhou, 310058 Zhejiang People’s Republic of China

**Keywords:** Tumor penetration, Extracellular matrix degradation, Biomimetic membrane, Photothermal therapy (PTT), Photodynamic therapy (PDT), CT imaging, Pancreatic cancer

## Abstract

**Background:**

Excessive extracellular matrix (ECM) deposition in pancreatic ductal adenocarcinoma (PDAC) severely limits therapeutic drug penetration into tumors and is associated with poor prognosis. Collagen is the most abundant matrix protein in the tumor ECM, which is the main obstacle that severely hinders the diffusion of chemotherapeutic drugs or nanomedicines.

**Methods:**

We designed a collagenase-functionalized biomimetic drug-loaded Au nanoplatform that combined ECM degradation, active targeting, immune evasion, near-infrared (NIR) light-triggered drug release, and synergistic antitumor therapy and diagnosis into one nanoplatform. PDAC tumor cell membranes were extracted and coated onto doxorubicin (Dox)-loaded Au nanocages, and then collagenase was added to functionalize the cell membrane through lipid insertion. We evaluated the physicochemical properties, in vitro and in vivo targeting, penetration and therapeutic efficacy of the nanoplatform.

**Results:**

Upon intravenous injection, this nanoplatform efficiently targeted the tumor through the homologous targeting properties of the coated cell membrane. During penetration into the tumor tissue, the dense ECM in the PDAC tissues was gradually degraded by collagenase, leading to a looser ECM structure and deep penetration within the tumor parenchyma. Under NIR irradiation, both photothermal and photodynamic effects were produced and the encapsulated chemotherapeutic drugs were released effectively, exerting a strong synergistic antitumor effect. Moreover, this nanoplatform has X-ray attenuation properties that could serve to guide and monitor treatment by CT imaging.

**Conclusion:**

This work presented a unique and facile yet effective strategy to modulate ECM components in PDAC, enhance tumor penetration and tumor-killing effects and provide therapeutic guidance and monitoring.

**Graphical Abstract:**

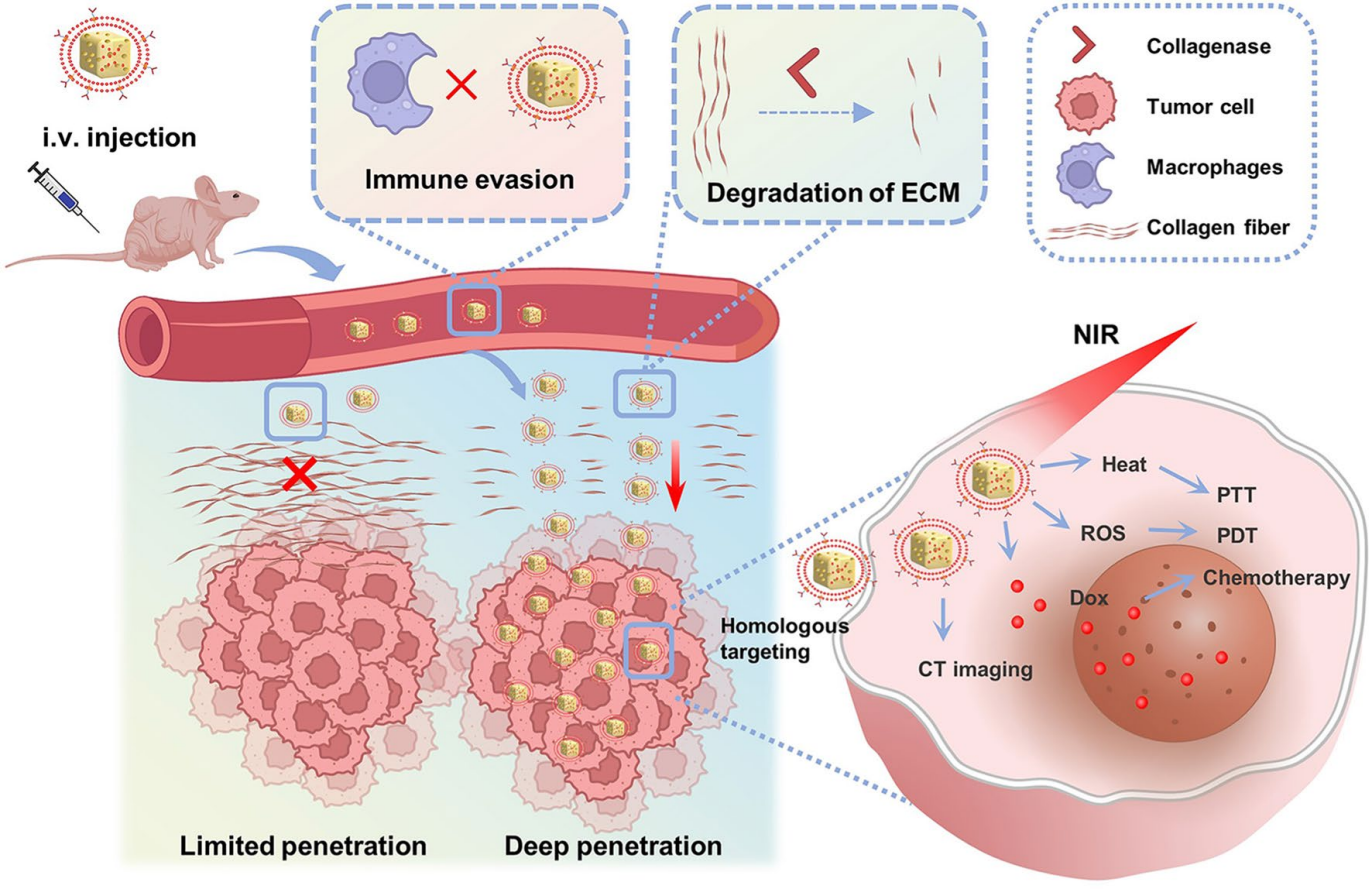

**Supplementary Information:**

The online version contains supplementary material available at 10.1186/s12951-022-01738-6.

## Background

Pancreatic ductal adenocarcinoma (PDAC) is one of the most lethal malignancies worldwide, with an overall 5 year survival of less than 10% [1–3]. Approximately 80–85% of patients present with either unresectable or metastatic disease and are no longer eligible for surgical resection at the time of diagnosis [4]. Therefore, treatment for most PDAC patients is limited to chemotherapy. Nevertheless, conventional chemotherapy has a very limited effect on improving outcomes, and the clinical prognosis remains poor [5, 6]. One of the primary causes of the unsatisfactory prognosis is the presence of a dense fibrotic stroma, which is commonly accompanied by excessive extracellular matrix (ECM) deposition [7]. This dense ECM produces solid stress and increases interstitial fluid pressure, which severely impedes the diffusion of drugs into tumor tissues, especially macromolecular drugs such as nanomedicines [6, 8].

Nanomedicines can exhibit better accumulation in tumors than conventional chemotherapeutic agents through the enhanced permeability and retention (EPR) effect [9, 10]. The FDA has approved Onivyde (liposomal irinotecan) and Abraxane (nab-paclitaxel) for PDAC treatment, both of which lead to an improvement in overall survival of only ~ 2 months [11–13]. Insufficient accumulation of these nanomedicines in tumor tissues is considered to be the main reason for their limited clinical efficacy [11]. These nanomedicines rely heavily on the EPR effect for passive tumor targeting and accumulation; however, this effect occurs to only a limited extent in PDAC due to the desmoplastic ECM. The abundant ECM components in the tumor microenvironment and their reticular structure prevent therapeutic drugs from entering tumor tissues. The regulation and remodeling of the tumor microenvironment has attracted considerable research interest in recent years [14, 15]. Various strategies have been developed to modulate the tumor ECM in the tumor microenvironment to reduce the hindrance of obstacles [16–18]. Notably, blocking ECM biogenesis is a widely studied strategy [18–20]. Although this approach can be used to fundamentally block the generation of ECM, it does not affect the existing ECM components, and diffusion efficiency is usually still insufficient. Another more direct and rapid approach is to modulate the ECM using ECM-degrading enzymes. Collagens are the most abundant matrix proteins in the tumor ECM, which presents the main obstacle that substantially hinders the diffusion of chemotherapeutic drugs or nanomedicines [21, 22]. Collagenase is an efficient ECM-degrading enzyme. Multiple studies have found that intravenous injection of collagenase into tumor-bearing mice can reduce collagen density and enhance the transport of the second wave of macromolecules or liposomes into tumors [23, 24]. However, circulating collagenase can damage normal tissues [25]. Moreover, first using collagenase followed by the second wave treatment of chemotherapy or nanomedicine is cumbersome and may lead to unnecessary collagen degradation in diffusion pathways unrelated to the nanoparticle. Thus, developing a collagenase-functionalized therapeutic nanoplatform that can not only reduce the matrix barrier but also exert strong therapeutic effects may be an attractive and promising approach for ECM modulation and enhanced PDAC treatment.

Therefore, an ideal collagenase-functionalized nanosystem should be able to combine collagenase to degrade the ECM in PDAC tissues and simultaneously effectively kill tumor cells. Au nanocages (AuNCs) represent a class of nanomaterials with tunable optical properties in the near-infrared (NIR) region and excellent stability and biocompatibility. AuNCs possess a photothermal therapy (PTT) effect and are widely applied in the thermal ablation of tumors due to their local surface plasmon resonance (LSPR) effect, which allows them to absorb light with a large cross-section and then efficiently convert the light into heat [26]. Additionally, under NIR irradiation, the excited state photon energy of AuNCs can transfer to molecular oxygen and generate cytotoxic reactive oxygen species (ROS) for a photodynamic therapy (PDT) effect [27–29]. In addition, the unique hollow porous structure of AuNCs makes them very suitable for drug encapsulation and controllable drug release through the photothermal effect of NIR light. In addition to favorable therapeutic efficiency, AuNCs possess a high X-ray attenuation coefficient, making them popular candidates for CT imaging [30–32]. Therefore, AuNCs are expected to be combined with collagenase to effectively degrade the ECM of PDAC tissues and achieve multidirectional tumor therapy. However, due to the hollow holes of AuNCs, chemotherapeutic drugs may leak out before they reach their target. Moreover, the effect of a nanoplatform targeting tumor tissues by the EPR effect is limited. In addition, how to conveniently connect collagenase to the AuNC platform is a problem that needs to be explored and solved. In this context, designing an actively targeted nanoplatform that can link collagenase and enable AuNC to encapsulate drugs is urgently needed.

**Scheme 1.**
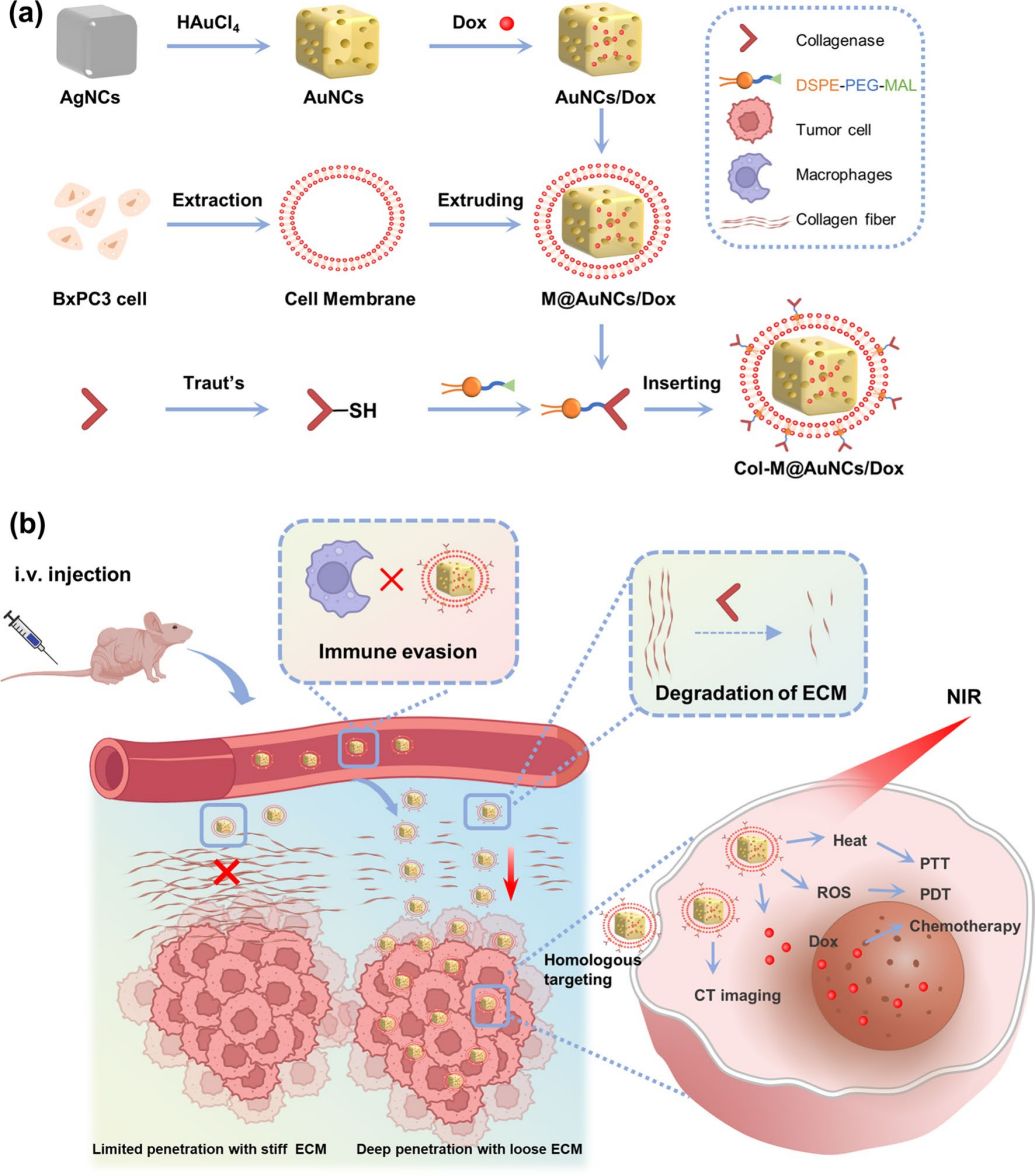
Schematic diagram. **a** Preparation of Col-M@AuNCs/Dox. **b** Col-M@AuNCs/Dox enhance intratumoral penetration and whole tumor destruction through ECM degradation and combined treatment and monitoring

Here, we rationally designed a nanosystem based on AuNCs to facilitate the degradation of the matrix barrier by collagenase and the treatment of PDAC through PTT, PDT and chemotherapy. First, we utilized AuNCs as versatile platforms for the delivery of the chemotherapeutic drug doxorubicin (Dox) and the achievement of PTT and PDT under NIR irradiation. Then, to overcome the problems of drug leakage and insufficient targeting and to easily link collagenase to AuNCs, we cleverly used biomimetic cell membrane coating technology, which involves coating a synthetic nanoparticle core with a natural cell membrane [33, 34]. Membrane-coated nanoparticles inherently mimic the properties of their membrane-derived cells, endowing nanoparticles with a wide range of functions, such as long circulation times and active targeting [35]. Cell membranes derived from tumor cells are capable of homologous tumor cell targeting and the prevention of immune clearance [36–38]. Therefore, we extracted PDAC tumor cell membranes and coated them onto the surface of Dox-loaded AuNCs, giving the AuNCs an active targeting function and immune evasion properties while reducing drug leakage [39]. Finally, based on the lipid bilayer properties of the cell membrane, we were able to conjugate collagenase by lipid insertion [40, 41] to eventually obtain collagenase-functionalized biomimetic Dox-loaded AuNCs(Col-M@AuNCs/Dox) (Scheme 1a). We hypothesized that the administered Col-M@AuNCs/Dox could efficiently target PDAC through homologous targeting. During infiltration from the extracellular stroma into the deep tumor tissue, the dense ECM in PDAC tissues was gradually degraded by collagenase so that the drug-carrying AuNCs could effectively penetrate tumor tissues. After the uptake of drug-loaded AuNCs by tumor cells, the combined effects of PTT, PDT and chemotherapy were exerted under irradiation with an 808 nm NIR laser. Moreover, the CT imaging performance of these AuNCs facilitated therapeutic guidance and monitoring (Scheme 1b). Collectively, the nanosystem we established overcomes the dense ECM barrier of PDAC tissue, facilitates the deep penetration of nanomedicines, and simultaneously potently enables combination therapy and monitoring, providing an efficacious approach to the dilemma of PDAC treatment. This ECM modulation strategy may also be expected to be applied to other tumor types with dense matrix to solve the problem of poor drug penetration.

## Results and discussion

### Preparation and characterization of Col-M@AuNCs/Dox

**Fig. 1:**
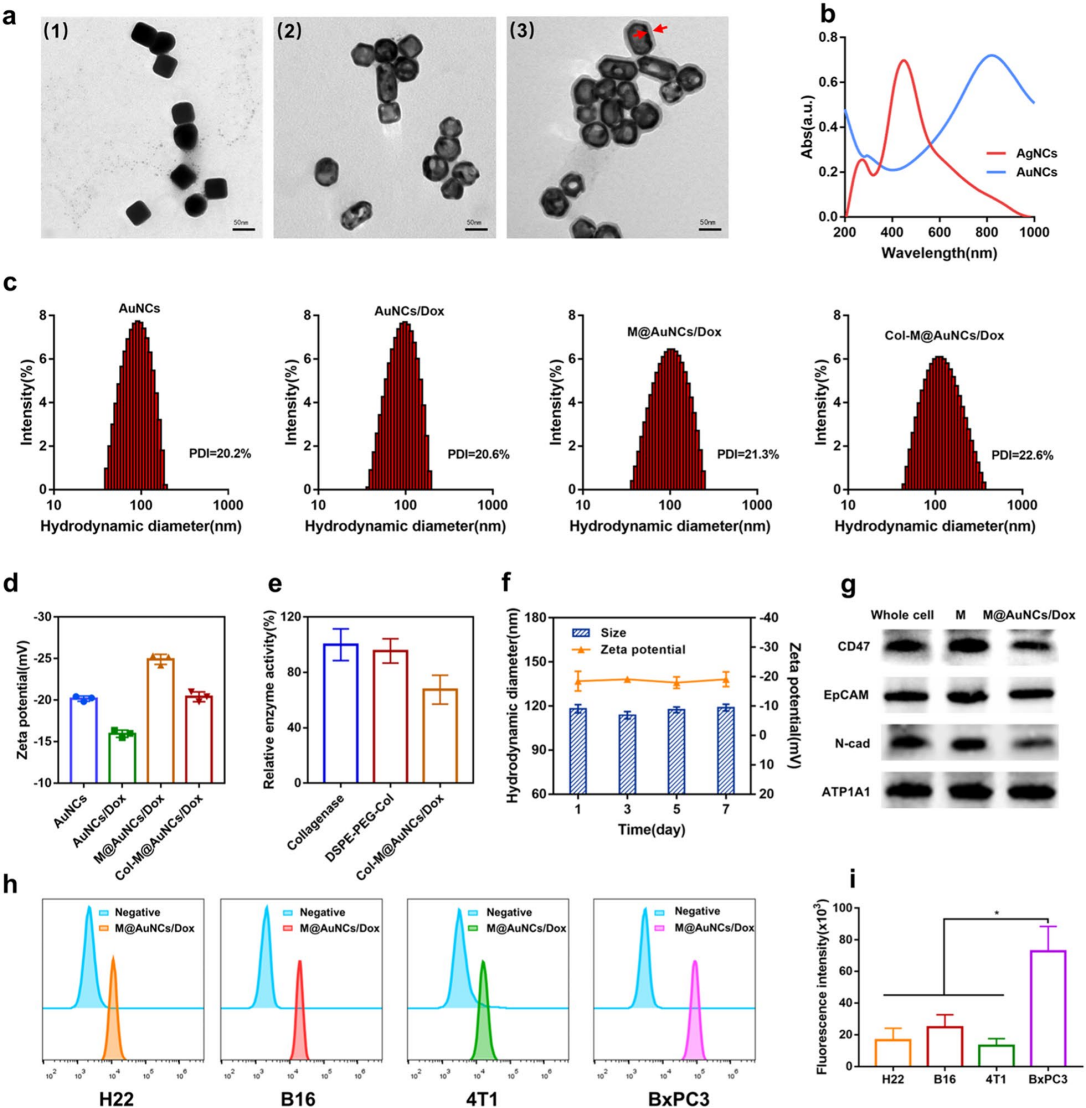
Characterization of Col-M@AuNCs/Dox. **a** TEM images of AgNCs (1), AuNCs (2) and M@AuNCs/Dox (3). **b** UV‒Vis absorption spectra of AgNCs and AuNCs. **c** Hydrodynamic diameters and polydispersity index (PDI) of AuNCs, AuNCs/Dox, M@AuNCs/Dox and Col-M@AuNCs/Dox. **d** Zeta potentials of AuNCs, AuNCs/Dox, M@AuNCs/Dox and Col-M@AuNCs/Dox. **e** Relative enzyme activities of free collagenase, DSPE-PEG-Col and Col-M@AuNCs/Dox. **f** Hydrodynamic diameter and zeta potential stability of Col-M@AuNCs/Dox. **g** Western blotting analysis of the proteins from BxPC3 cells, BxPC3 cell membranes and M@AuNCs/Dox. **h**–**i** FCM analysis of the cellular uptake of M@AuNCs/Dox into H22, B16, 4T1 and BxPC3 cells and the quantitative analysis of fluorescence intensity

AuNCs were synthesized by the galvanic replacement reaction between Ag nanocubes (AgNCs) and HAuCl_4_. Solid AgNCs were first synthesized as shown in the transmission electron microscopy (TEM) image in Fig. 1a(1), and then hollow porous AuNCs were obtained (Fig. 1a(2)) using AgNCs as templates. The observed absorption peak migrated from 400 to 800 nm (Fig. 1b), indicating the successful synthesis of AuNCs with characteristic LSPR in the NIR region, which is an important physicochemical characteristic of AuNCs to exert PTT and PDT effects. The average hydrodynamic diameter of the AuNCs was approximately 88 nm (Fig. 1c), and their surface zeta potential was − 20.2 mV (Fig. 1d). Next, Dox was loaded into the AuNCs by electrostatic attraction to obtain drug-loaded AuNCs/Dox, and the drug loading capacity and encapsulation rate were 2.66 ± 0.15% and 54.7 ± 3.06%, respectively. The hydrodynamic diameter of AuNCs/Dox was 94.4 nm (Fig. 1c), and the zeta potential was − 15.9 mV (Fig. 1d). The extrusion method was used to coat the cell membrane on the surface of AuNCs/Dox, and the morphology of the obtained M@AuNCs/Dox is shown in Fig. 1a (3). M@AuNCs/Dox exhibited a core–shell structure with an AuNC core enclosed in a thin, smooth membrane shell. A slight increase in hydrodynamic diameter (104 nm, Fig. 1c) and decrease in zeta potential (− 24.9 mV, Fig. 1d) were detected after the cell membrane coating. Both the particle size and zeta potential changes indicated that AuNCs/Dox were successfully coated with the cell membrane.

Finally, the membrane surface was functionalized with collagenase by lipid insertion. This method relies on physical attachment in which the lipid component of the biomolecule is inserted into the lipid bilayer of the membrane. This process is convenient and simple and does not require the biomembranes to be exposed to any chemical reactions, which helps preserve the integrity of the cell membrane proteins [40]. First, the maleimide of 1,2-Distearoyl-sn-glycero-3-phosphoethanolamine-N-[maleimide(polyethyleneglycol)] (DSPE-PEG-MAL) was reacted with the sulfhydryl group on SH-Col to obtain the DSPE-PEG-Col lipid-collagenase conjugates. ^1^H NMR spectroscopy was used to characterize the successful synthesis of DSPE-PEG-Col. As shown in Additional file 1: Fig. S1, δ = 6.73 ppm was the proton peak of maleimide, and δ = 3.56 ppm was the proton peak of the PEG chain. Both peaks could be observed in DSPE-PEG-MAL. In the product DSPE-PEG-Col, the maleimide peak disappeared, indicating that it had successfully reacted with the sulfhydryl group. The next step was to use the lipophilic properties of DSPE to insert DSPE-PEG-Col into the lipid bilayer of the cell membrane, which is often used for the surface functionalization of liposomes. Since cell membranes and liposomes have similar lipid bilayer properties, we used this method to connect collagenase to the surface of the cell membranes. The final obtained collagenase-functionalized biomimetic Dox-loaded Au nanocages (Col-M@AuNCs/Dox) had a particle size of 114.6 nm (Fig. 1c) and a surface zeta potential of − 20.4 mV (Fig. 1d). The binding efficiency of collagenase was 24.02 ± 4.59% and the binding amount of collagenase on nanoparticles was calculated as 217 ng per 10^11^ particles. Collagenase was linked to the prepared M@AuNCs/Dox to enhance its tumor penetration ability based on the degradation of the dense collagen surrounding tumor cells. Due to the homology of gelatin and collagen, the enzyme activity of Col-M@AuNCs/Dox was measured by examining gelatin degradation (Additional file 1: Fig. S2). Pure gelatin (30 mg/mL) was dissolved in warm water, but resolidified into a hydrogel at low temperature. In addition, stable hydrogels were still formed at 4 °C after coincubation with M@AuNCs/Dox, indicating that gelatin could not be degraded. However, the gelatin remained in liquid form after coincubation with free collagenase, DSPE-PEG-Col and Col-M@AuNCs/Dox, indicating that the gelatin was degraded into low molecular weight products. We further accurately quantified the collagenase activity changes using a collagenase enzyme activity detection kit. The final Col-M@AuNCs/Dox collagenase activity was approximately 67.5% that of the original free collagenase (Fig. 1e), indicating a slight decrease in collagenase activity after nanosystem synthesis.

Considering the colloidal stability, different preparations in PBS containing 10% FBS were placed at room temperature for 1 week. AuNCs and AuNCs/Dox precipitated slightly, while the M@AuNCs/Dox and Col-M@AuNCs/Dox solutions retained good dispersity (Additional file 1: Fig. S3), showing that the membrane coating was helpful to maintain the stability of the nanosystem. The hydrodynamic diameter and zeta potential of Col-M@AuNCs/Dox were recorded and showed no notable changes over 7 days (Fig. 1f).

### Characterization of the membrane proteins on M@AuNCs/Dox and their targeting ability

To verify the retention of the membrane proteins on M@AuNCs/Dox, Sodium dodecyl sulfate–polyacrylamide gel electrophoresis (SDS‒PAGE) was carried out. Protein electrophoresis indicated the presence of BxPC3 cell membrane proteins on the M@AuNCs/Dox (Additional file 1: Fig. S4), suggesting successful integration of the cell membrane and AuNCs and retention of the membrane proteins. It has been reported that certain proteins on cell membranes contribute to immune evasion and homologous recognition, including CD47, EpCAM, and N-cadherin [33, 42]. CD47 helps tumor cells escape recognition of the immune system by avoiding phagocytosis by mononuclear macrophages. EpCAM and N-cadherin are involved in tumor cell adhesion and the recognition of targets. Therefore, Western blotting was further used to evaluate the retention of these functional proteins on M@AuNCs/Dox. As shown in Fig. 1g, these functional proteins were successfully retained on M@AuNCs/Dox after coating. Na^+^/K^+^-ATPase (ATP1A1, a plasma membrane-specific marker) was also enriched on M@AuNCs/Dox, demonstrating successful retention of membrane proteins after the fabrication process.

The ability of M@AuNCs/Dox to target homologous BxPC3 cells was investigated in several cell types. After M@AuNCs/Dox were coincubated with H22, B16, 4T1 and BxPC3 cells, the intracellular Dox fluorescence intensity was detected by flow cytometry (FCM). The intracellular fluorescence intensity of BxPC3 cells was significantly higher than that of other cell lines (Fig. 1h, i), suggesting that membrane encapsulation specifically enhanced uptake by homologous tumor cells.

To further study the immune escape ability of M@AuNCs/Dox, AuNCs/Dox and M@AuNCs/Dox were coincubated with macrophage-like THP-1 cells. The intracellular Dox fluorescence intensity of THP-1 cells was detected by FCM, and the results are shown in Additional file 1: Fig. S5. The uptake of M@AuNCs/Dox into THP-1 cells was less than the uptake of AuNCs/Dox, indicating that the membrane coating reduced the macrophage uptake of the nanosystem. The low internalization observed in macrophages may have been attributed to the high expression of CD47 on M@AuNCs/Dox, which promotes the evasion of phagocytosis [43].

### In vitro* PTT, PDT and CT imaging*

**Fig. 2:**
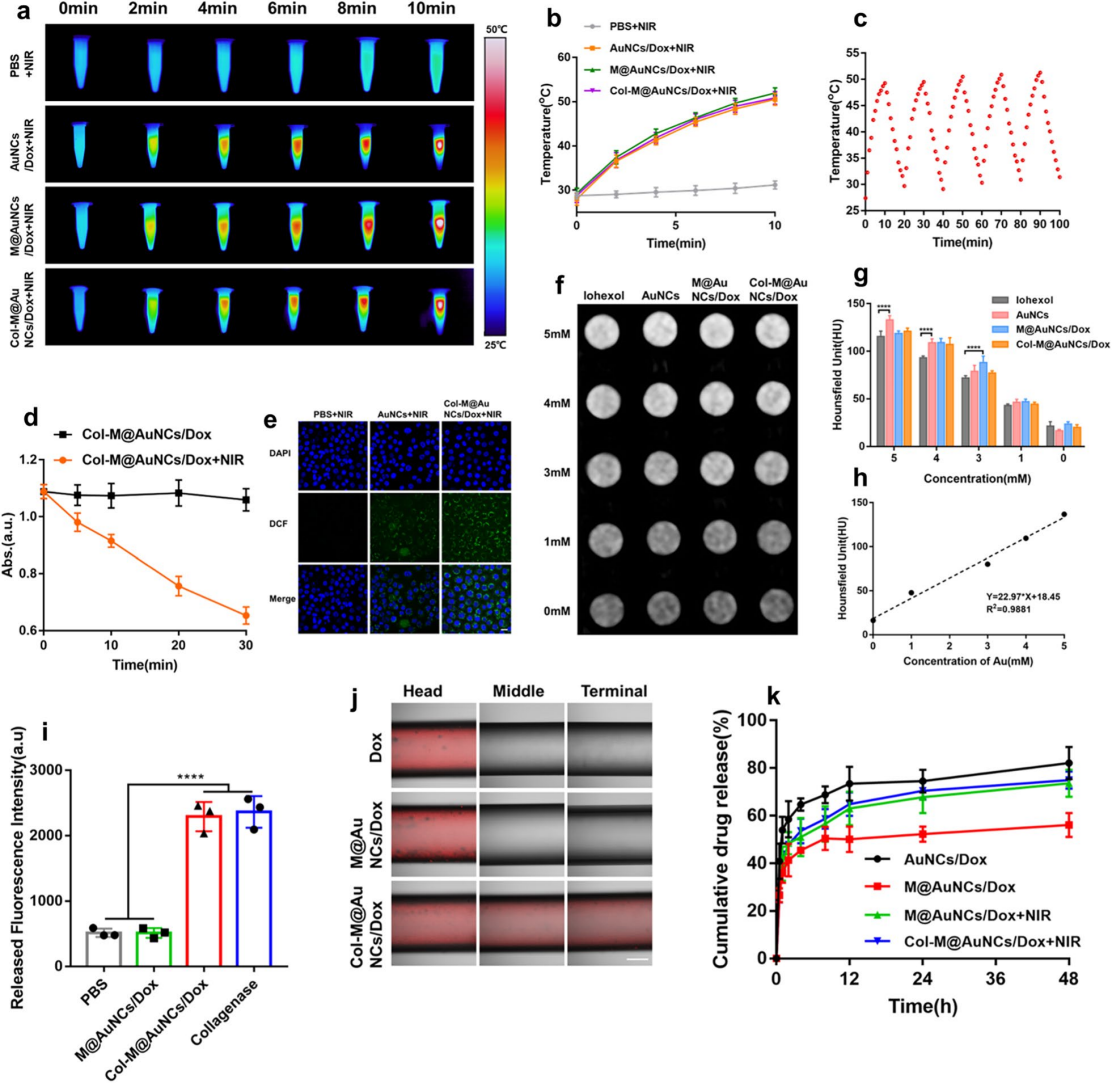
PTT, PDT, and CT imaging effects, collagen degradation and NIR-triggered drug release of Col-M@AuNCs/Dox.** a** Infrared thermal images and **(b)** temperature curves under NIR irradiation. **c** Photothermal stability of Col-M@AuNCs/Dox after five cycles of irradiation and cooling. **d** ROS generation by Col-M@AuNCs/Dox upon NIR irradiation using DPBF as the probe. **e** CLSM observation of ROS detection in BxPC3 cells treated with AuNCs or Col-M@AuNCs/Dox with NIR irradiation (scale bar: 20 μm). **f** CT images and **(g)** quantitative CT values of AuNCs, M@AuNCs/Dox, Col-M@AuNCs/Dox and iohexol at varying Au or I concentrations of 0–5 mM. **h** CT linear fitting values of Col-M@AuNCs/Dox. **i** Fluorescein released by a FITC-containing collagen matrix treated with PBS, M@AuNCs/Dox, Col-M@AuNCs/Dox and collagenase. **j** M@AuNCs/Dox and Col-M@AuNCs/Dox diffusion into ECM-mimicking gels in capillary tubes (scale bar: 200 μm). **k** Dox release profiles from AuNCs/Dox, M@AuNCs/Dox and Col-M@AuNCs/Dox with and without NIR irradiation

The PTT and PDT effects and CT imaging characteristics of the nanosystem were further explored. Due to the LSPR characteristics of AuNCs, a high temperature can be generated under irradiation with an 808 nm laser, and the temperature rise was linearly related to the concentration and laser power (Additional file 1: Fig. S6). Clearly, Col-M@AuNCs/Dox treatment led to hyperthermia even at a low concentration (30 μg/mL), which is important to eliminate cancer cells (requiring a temperature above 42 °C). Thermographic images after irradiation with an 808 nm laser (1 W/cm^2^) for different lengths of time were captured (Fig. 2a). After 10 min of irradiation, the temperature of PBS increased by only 2 °C. However, AuNCs/Dox, M@AuNCs/Dox and Col-M@AuNCs/Dox showed similar heating curves (Fig. 2b), as the temperature increased by approximately 22 °C, reaching a high temperature of 50 °C. The photothermal stability of Col-M@AuNCs/Dox was further tested. After five cycles of irradiation, no significant changes in PTT performance were observed (Fig. 2c), demonstrating the desirable PTT stability of Col-M@AuNCs/Dox.

The PDT effect of the Col-M@AuNCs/Dox solution was explored using the chemical trapping reagent 1,3-Diphenylisobenzofuran (DPBF) as a probe, which can react with ROS to form an endoperoxide product, resulting in a decrease in the absorbance of the characteristic absorption peak (410 nm)[27]. As shown in Fig. 2d, the absorbance of DPBF was almost unchanged in the absence of laser irradiation. However, under NIR laser irradiation, the absorbance of DPBF continued to decline, indicating that Col-M@AuNCs/Dox produced ROS under NIR irradiation and consumed DPBF. These results showed that Col-M@AuNCs/Dox can effectively produce ROS under NIR laser exposure.

Next, the PDT effect of Col-M@AuNCs/Dox was further explored in BxPC3 cells using 2',7'-dichlorodihydrofluorescein diacetate (DCFH-DA) as a probe, which reacts with ROS and is converted into fluorescent DCF, and the confocal laser scanning microscopy (CLSM) results are shown in Fig. 2e. No DCF fluorescence was observed in the PBS group under illumination. Col-M@AuNCs/Dox + NIR-treated cells showed notable green fluorescence that was stronger than the fluorescence in AuNCs + NIR-treated cells. Since the homologous targeting ability of tumor cell membranes contributes to increased Col-M@AuNCs/Dox cellular uptake, the improved PDT effect might have been related to the more effective cellular uptake of Col-M@AuNCs/Dox. The FCM results quantitatively confirmed the above findings (Additional file 1: Fig. S7), indicating that Col-M@AuNCs/Dox had good PDT effects in cells.

Due to the high atomic number (79) and high density of Au, AuNCs may possess favorable X-ray attenuation properties. As shown in Fig. 2f, g, compared with the clinical CT contrast agent iohexol, AuNCs had great CT attenuation performance. In addition, M@AuNCs/Dox and Col-M@AuNCs/Dox had attenuation effects similar to those of AuNCs, showing that the cell membrane and collagenase had no significant effect on the CT imaging effect of the AuNCs. The CT values of Col-M@AuNCs/Dox increased linearly with increasing concentration (Fig. 2h). Collectively, Col-M@AuNCs/Dox demonstrated favorable PTT, PDT and CT imaging effects in vitro, which laid a good foundation for further application of Col-M@AuNCs/Dox in combination therapy and diagnosis.

### *Collagen degradation behavior *in vitro

Collagen gel containing FITC was used to study the collagen degradation efficacy of Col-M@AuNCs/Dox. When the gel was treated with collagenase, the FITC within the gel was released due to collagen degradation. Thus, the fluorescence intensity in the supernatant can reflect the efficacy of collagen degradation of the preparation, as shown in Fig. 2i. The supernatant of the PBS and M@AuNCs/Dox samples had only slight fluorescence intensity, which may have been due to a small amount of FITC present on the gel surface. In contrast, the fluorescence intensity of Col-M@AuNCs/Dox was significantly higher than that of the PBS and M@AuNCs/Dox samples, showing that Col-M@AuNCs/Dox can effectively degrade collagen gel and release FITC. In addition, the FITC fluorescence intensity released in the free collagenase-treated gels was similar to that in the Col-M@AuNCs/Dox-treated gels, indicating that the degradation effect of Col-M@AuNCs/Dox in collagen gels was similar to that of free collagenase.

The collagen degradation effect of Col-M@AuNCs/Dox was further studied with a capillary diffusion test. Collagen gel was loaded into the capillary, and different preparations were added to the head of the capillary. Finally, Dox fluorescence at the head, middle and tail of the capillary was observed (Fig. 2j). In the free Dox and M@AuNCs/Dox groups, Dox fluorescence existed in only the head of the capillary but did not penetrate the center and tail. However, in the Col-M@AuNCs/Dox group, Dox fluorescence was detected in the head, center and tail of the capillary, indicating that Col-M@AuNCs/Dox degraded the collagen gel and changed the gel into a liquid form, thus allowing Col-M@AuNCs/Dox to penetrate the capillary and Dox fluorescence to be observed in the center and tail. The results indicated that Col-M@AuNCs/Dox can effectively degrade collagen in an in vitro ECM model.

### On-demand NIR-induced drug release of Col-M@AuNCs/Dox

Dynamic dialysis was used to conduct drug release experiments, and the results are shown in Fig. 2k. Within 48 h, 82.1% of the drug was released from AuNCs/Dox, while 56.1% of the drug was released by the M@AuNCs/Dox, suggesting that the membrane coating reduced drug leakage. Next, the effect of NIR illumination on drug release was observed. Drug release reached 73.6% 48 h after M@AuNCs/Dox underwent NIR illumination. The drug release curves of Col-M@AuNCs/Dox and M@AuNCs/Dox were almost the same, demonstrating that collagenase coupling had no significant effect on drug release. The results showed that the hyperthermia and ROS induced by NIR irradiation may increase the permeability of the coated membrane and significantly increase the release of the drug. Therefore, the cell membrane coating can effectively respond to the stimulation of NIR irradiation and achieve controllable drug release.

### Cell uptake and Dox intracellular release

**Fig. 3:**
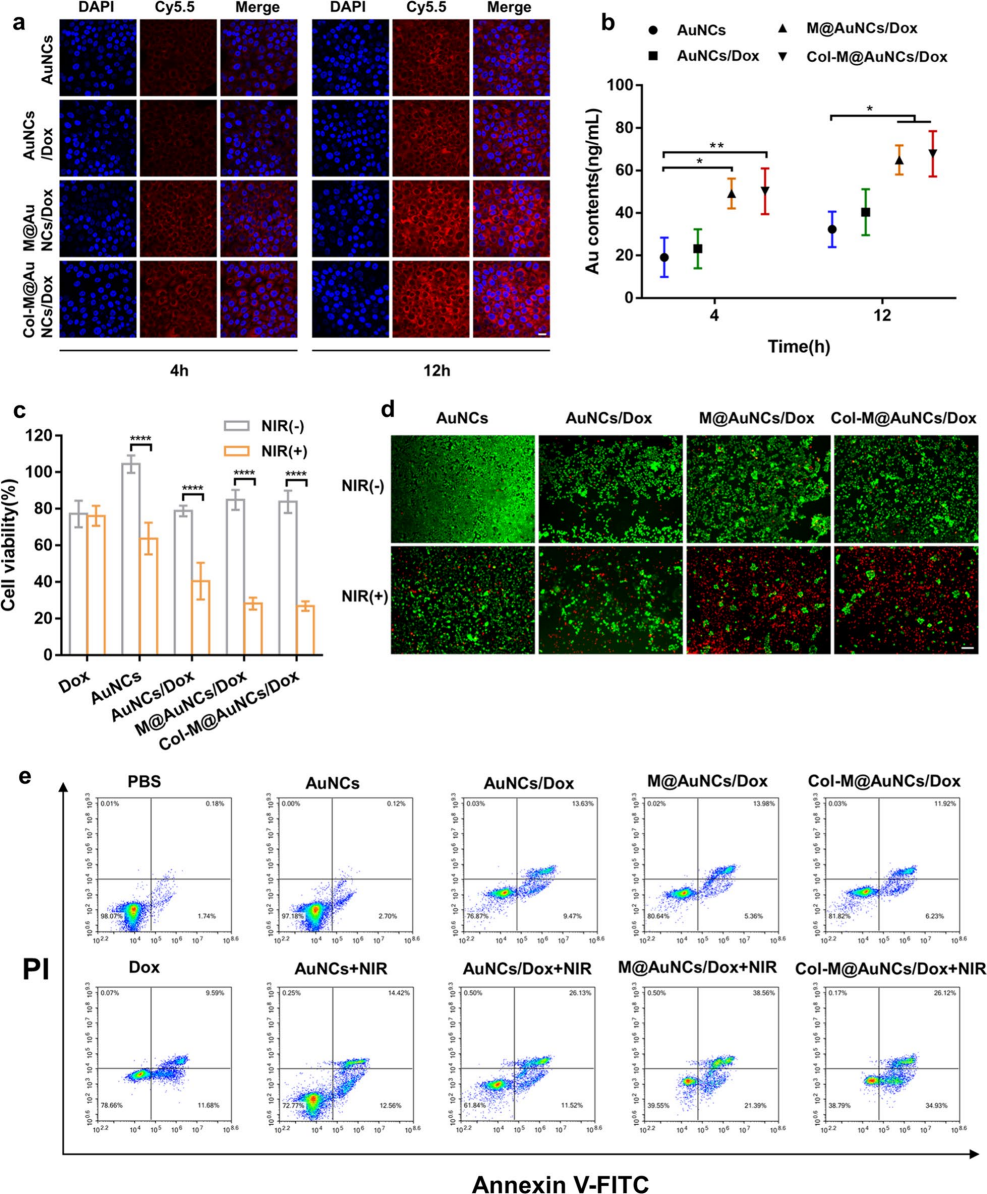
Cell uptake and cytotoxicity. **a** CLSM images of BxPC3 cells after incubation with Cy5.5-labeled AuNCs, AuNCs/Dox, M@AuNCs/Dox and Col-M@AuNCs/Dox for 4 and 12 h (scale bar: 20 μm). **b** ICP–MS analysis of the intracellular Au levels in BxPC3 cells after incubation with AuNCs, AuNCs/Dox, M@AuNCs/Dox and Col-M@AuNCs/Dox for 4 and 12 h. **c** Viability of BxPC3 cells treated with Dox, AuNCs, AuNCs/Dox, M@AuNCs/Dox and Col-M@AuNCs/Dox with or without NIR irradiation. **d** Live/dead staining images of BxPC3 cells after treatment with the different preparations. Live cells were stained with calcein-AM (green), and dead cells were stained with propidium iodide (red) (scale bar: 100 µm). **e** FCM analysis of the apoptosis levels of BxPC3 cells after incubation with different preparations with or without NIR irradiation

To study the BxPC3 cellular uptake efficiency of different preparations, cell uptake experiments were carried out with Cy5.5-labeled preparations. CLSM was used to observe the fluorescence of Cy5.5 in the cells to reflect the uptake efficiency of the nanosystem (Fig. 3a). The uptake of different preparations was time dependent, and there was only slight red fluorescence in AuNCs-treated cells. The fluorescence intensity in the AuNCs/Dox group was slightly higher than that in the AuNCs group, which may have been due to the fact that Dox loading attenuated the negative surface potential and promoted the absorption of the nanosystem into cells. The M@AuNCs/Dox-treated cells showed significantly higher fluorescence than AuNCs/Dox and AuNCs, indicating that the cell membrane coating effectively promoted the cellular uptake of the nanosystem. The Col-M@AuNCs/Dox group presented similar fluorescence to the M@AuNCs/Dox group. Since cells were cultured as a monolayer without ECM, the effect of collagenase could not be observed, indicating that the coupling of collagenase had little influence on cell uptake. Inductively coupled plasma-mass spectrometry (ICP‒MS) showed similar trends to those observed in CLSM, as shown in Fig. 3b. M@AuNCs/Dox and Col-M@AuNCs/Dox showed significantly higher cellular uptake than AuNCs/Dox and AuNCs. The 4 h-cell uptake in the M@AuNCs/Dox and Col-M@AuNCs/Dox groups was 2.6 times and 2.1 times that in the AuNCs and AuNCs/Dox groups, respectively, and the 12 h-cell uptake was 2 times and 1.6 times that in the AuNCs and AuNCs/Dox groups, respectively. Here, the enhancement at 12 h was less than that at 4 h, which may be due to the fact that the cell uptake in the M@AuNCs/Dox and Col-M@AuNCs/Dox groups was close to saturation at 12 h, resulting in a diminished enhancement effect. Overall, these results suggested that the membrane coating effectively enhanced the uptake of the nanosystem by BxPC3 cells.

To verify whether NIR-triggered drug release could be achieved in BxPC3 cells, the intracellular Dox distribution from the nanocarrier under NIR irradiation and non-NIR irradiation conditions was examined. Dox is a chemotherapy drug that acts in the nucleus, and free Dox can enter the nucleus, while encapsulated Dox cannot. Therefore, the NIR response characteristics of the preparation can be reflected by the intracellular distribution of Dox. As shown in Additional file 1: Fig. S8, in the absence of NIR irradiation, Dox fluorescence in the free Dox group was located in the nucleus, and the intensity was weak. The fluorescence of the AuNCs/Dox group was also mainly located in the nucleus, as AuNCs/Dox did not have a membrane coating, and the drug was quickly released from AuNCs and entered the nucleus. The Dox fluorescence in the M@AuNCs/Dox group and Col-M@AuNCs/Dox group was mainly located in the cytoplasm, indicating that Dox was loaded into the carrier and could not enter the nucleus as the free drug. However, the fluorescence in the M@AuNCs/Dox and Col-M@AuNCs/Dox groups was transferred from the cytoplasm to the nucleus after NIR irradiation, suggesting that NIR irradiation triggered the release of the drug from the M@AuNCs/Dox and Col-M@AuNCs/Dox systems, which then entered the nucleus. These results demonstrated that the membrane coating can respond well to NIR irradiation and achieve spatiotemporally controllable drug release.

### Cytotoxicity evaluation

The in vitro efficacy of the nanosystem was assessed using the MTT assay. The safety of AuNCs and collagenase was first evaluated. When the concentration of AuNCs reached 300 µg/mL, the cell viability was over 90%, indicating the good biocompatibility of the carrier (Additional file 1: Fig. S9a). Moreover, when the collagenase concentration reached 1000 µg/mL, the cell viability was also greater than 90%, suggesting that collagenase had no significant effect on cell viability at this concentration (Additional file 1: Fig. S9b). No significant difference was observed in Dox-treated cells with or without NIR irradiation, indicating that the NIR laser had no effect on Dox cytotoxicity (Fig. 3c). The cell viability of the AuNCs/Dox group was 78% in the absence of light, which was similar to that of free Dox. M@AuNCs/Dox and Col-M@AuNCs/Dox had a similar effect on cells, with 85% cell viability without NIR irradiation. After NIR irradiation, AuNCs displayed cytotoxicity with a cell viability of 64%, suggesting that the high temperature and ROS produced by AuNCs had a killing effect on the cells. Cell viability in the AuNCs/Dox + NIR group was 41%, suggesting that phototherapy combined with Dox chemotherapy can enhance the individual effects. In the M@AuNCs/Dox + NIR and Col-M@AuNCs/Dox + NIR groups, cell viability was similar (approximately 28%), indicating that the effect of the membrane coating to increase cell uptake significantly enhanced the efficacy of combined phototherapy and chemotherapy. Since monolayer cells without obvious ECM were used in the MTT assay, collagenase did not play a clear role. Cytotoxicity was visually observed by live/dead assays (Fig. 3d). In the M@AuNCs/Dox and Col-M@AuNCs/Dox groups, most cells died under NIR irradiation, indicating that M@AuNCs/Dox + NIR and Col-M@AuNCs/Dox + NIR could effectively kill cells through combined phototherapy and chemotherapy in the monolayer cell state. The apoptosis-induced effects of different preparations were further studied by apoptosis analysis (Fig. 3e), and the results showed a similar trend to those from the MTT assay. The M@AuNCs/Dox + NIR and Col-M@AuNCs/Dox + NIR groups showed significant apoptosis and necrosis.

Taken together, these data show that in in vitro monolayer cultured cells, M@AuNCs/Dox and Col-M@AuNCs/Dox had potent combined killing effects based on phototherapy and chemotherapy under NIR irradiation.

### Penetration and toxicity in multicellular tumor spheroids (MTSs)

**Fig. 4:**
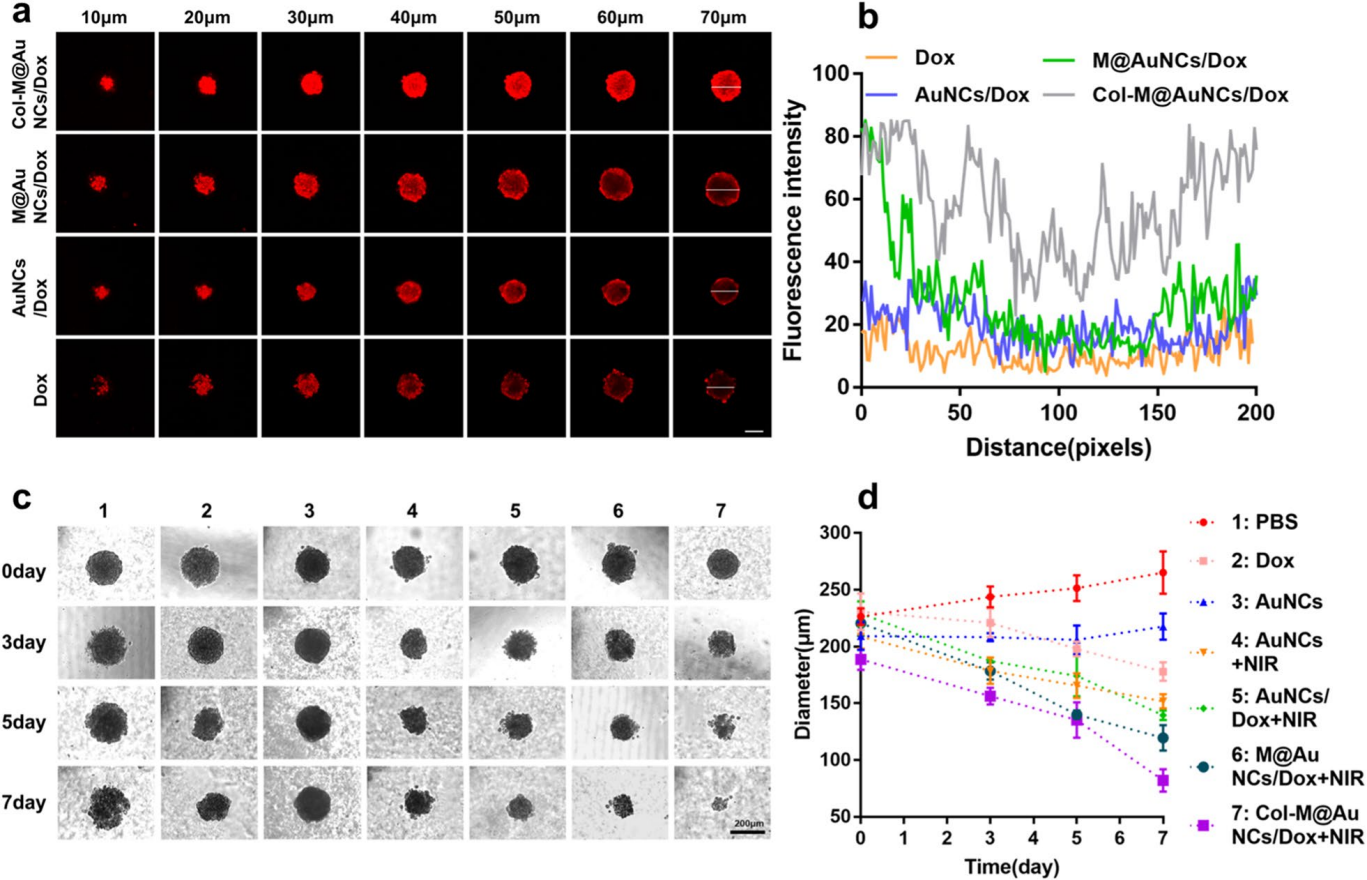
Penetration and toxicity in BxPC3 MTSs. **a** CLSM z-stack images of BxPC3 MTSs after incubation with Dox, AuNCs/Dox, M@AuNCs/Dox and Col-M@AuNCs/Dox (scale bar: 200 μm) and (**b**) the fluorescence intensity profiles along the selected lines. **c** Optical images and (**d**) diameters of BxPC3 MTSs treated with different formulations for 7 days

We explored the degradation and penetration of Col-M@AuNCs/Dox in collagen gel in vitro and the cellular uptake and cytotoxicity of Col-M@AuNCs/Dox in monolayer cells. To better simulate the ECM in tumors and better study the degradation and penetration effect of Col-M@AuNCs/Dox, we established a BxPC3 MTS model, which can more accurately simulate the growth status of tumor cells [44]. First, the penetration of different preparations was studied. As depicted in Fig. 4a, in the free Dox and AuNCs/Dox groups, there was only slight fluorescence on the outer layer of the MTSs, suggesting that the drug had difficulty penetrating deep into the MTSs. The fluorescence intensity in the M@AuNCs/Dox-treated MTSs was slightly stronger than that in Dox- and AuNCs/Dox-treated MTSs. Due to the cell membrane targeting effect, cell uptake was increased, but the fluorescence was also limited to the surface of the MTSs, indicating that M@AuNCs/Dox still had difficulty penetrating the MTSs. Col-M@AuNCs/Dox showed significantly stronger fluorescence than the other groups and almost penetrated the MTSs. This finding may indicate that the ECM became loose along the diffusion path of the nanosystem with the degradation of collagen, leading to smooth penetration of the nanosystem into the deep regions of the MTSs. Quantitative analysis of fluorescence intensity demonstrated that the Col-M@AuNCs/Dox group produced the most intense fluorescence in the middle area of the MTSs (Fig. 4b).

To further study the effects of the different preparations on the growth of MTSs, we observed the one-week growth of MTSs treated with different nanosamples with or without NIR irradiation. Figure 4c, d depicted that the MTSs in PBS and AuNCs groups grew normally. The volume of the MTSs in the free Dox group decreased slightly, indicating that Dox has a certain inhibitory effect. In the AuNCs + NIR group, the PTT and PDT effects produced under NIR irradiation also inhibited tumor growth to some extent. The inhibitory effect of the AuNCs/Dox + NIR group on MTSs growth was slightly better than that of the Dox and AuNCs + NIR groups, suggesting the enhanced effect of combined phototherapy and chemotherapy. The effect in the M@AuNCs/Dox + NIR group was also better than that in the AuNCs/Dox + NIR group, again indicating that the enhanced cellular uptake promoted the killing effect. The final preparation, the Col-M@AuNCs/Dox + NIR group, showed the best inhibitory effect. After seven days, the MTS volume decreased significantly, and the MTSs nearly disintegrated and disappeared. These results demonstrated that with the help of ECM degradation by collagenase, the nanosystem effectively penetrated the MTSs and released chemotherapy drugs under NIR irradiation, and also carried out both PTT and PDT to kill the MTSs, resulting in the maximum killing effect.

### In vivo* biodistribution*

**Fig. 5:**
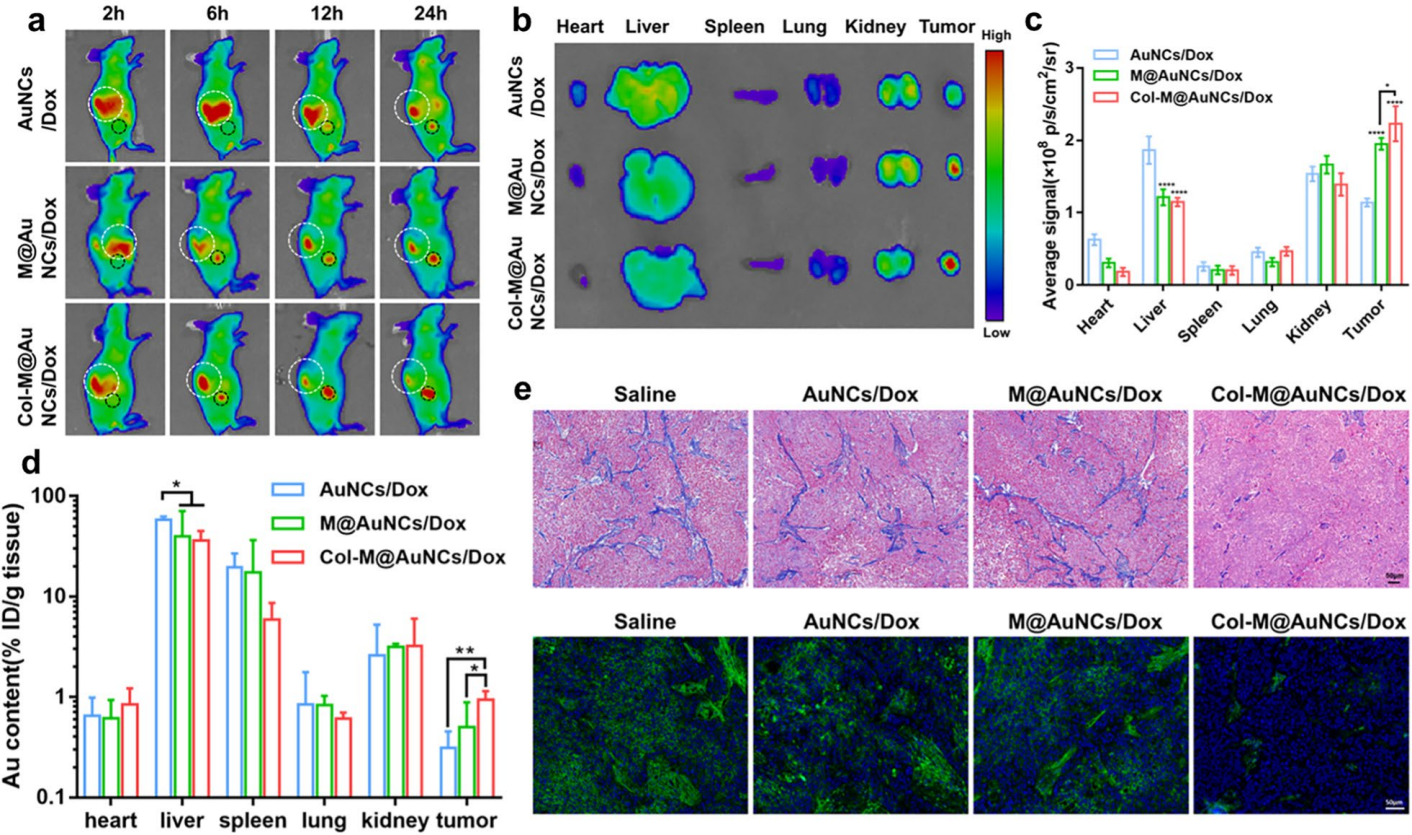
In vivo biodistribution and collagen degradation effect. **a** In vivo fluorescence images of BxPC3 tumor-bearing, mice after intravenous injection of Cy5.5-labeled AuNCs/Dox, M@AuNCs/Dox and Col-M@AuNCs/Dox. **b** Ex vivo fluorescence images of the major organs and tumors excised from mice 24 h after injection. **c** Average radiant efficiency of the excised organs and tumors. **d** ICP‒MS analysis of the Au levels in major organs and tumors excised from the mice 24 h after injection. **e** Masson’s trichrome analysis (collagen fibers are blue) and immunofluorescence images of collagen I (green) in tumor slices collected from mice receiving AuNCs/Dox, M@AuNCs/Dox and Col-M@AuNCs/Dox

Based on the potent tumor cell killing effect of Col-M@AuNCs/Dox in vitro, we next carried out further studies in BxPC3 tumor-bearing mice. First, Cy5.5-labeled nanoplatforms were used to study the biological distribution of the different preparations in vivo, and the results are shown in Fig. 5a. In the AuNCs/Dox group, there was no obvious drug accumulation in the tumor (black circle) at 2–6 h, while the drug showed obvious fluorescence in the liver (white circle). At 12–24 h, there was slight fluorescence aggregation at the tumor site. Fluorescence accumulation began in the M@AuNCs/Dox group at 6 h and gradually increased from 12 to 24 h, which was stronger than the AuNCs/Dox group, indicating that the targeting effect of the cell membrane enhanced the accumulation of the nanosystem in the tumor. Moreover, the fluorescence intensity in the livers of the mice in the M@AuNCs/Dox group was significantly lower than that in the AuNCs/Dox group, indicating that the cell membrane coating reduced the retention of the nanosystem by the liver (with the abundant accumulation of macrophages) [45, 46]. The Col-M@AuNCs/Dox group presented significantly higher tumor fluorescence intensity than the AuNCs/Dox group, indicating that by degrading collagenase, the nanocarrier continuously penetrated and efficiently accumulated in the tumor tissue. In addition, the liver uptake of Col-M@AuNCs/Dox was similar to that of M@AuNCs/Dox, indicating that the cell membrane coating can still reduce the uptake of the nanosystem by the liver. The mice were dissected at 24 h for ex vivo fluorescence intensity analysis and quantification, and the results are shown in Fig. 5b, c. The tumor fluorescence in the mice treated with AuNCs/Dox was lower than that in M@AuNCs/Dox and Col-M@AuNCs/Dox groups, but the fluorescence intensity of liver was stronger than that in M@AuNCs/Dox and Col-M@AuNCs/Dox groups. Furthermore, the tumor fluorescence in Col-M@AuNCs/Dox group was higher than that in the M@AuNCs/Dox group, and the fluorescence in the liver was similar to that in the M@AuNCs/Dox group.

Moreover, the dissected tumors and organs were digested with aqua regia, and their Au contents were quantitatively analyzed by ICP‒MS to further explore the biological distribution of the nanosystem in vivo. Figure 5d depicted a trend similar to the results of the ex vivo fluorescence quantitative analysis. The maximum tumor accumulation occurred in the Col-M@AuNCs/Dox group, while the distribution of Col-M@AuNCs/Dox in the liver was similar to that of M@AuNCs/Dox and significantly lower than that of AuNCs/Dox. These results indicated that in the BxPC3 tumor-bearing mouse model, Col-M@AuNCs/Dox had an effective tumor targeting ability and higher intratumor accumulation.

### In vivo* collagen degradation effect and tumor penetration*

To study the effect of Col-M@AuNCs/Dox on tumor ECM in vivo, Masson and collagen immunofluorescence staining were performed on tumor slices, and the results are shown in Fig. 5e. In the saline-, AuNC/Dox- and M@AuNC/Dox-treated groups, Masson and immunofluorescence staining showed that the tumor tissue contained abundant collagen fibers. In the Col-M@AuNC/Dox-treated group, the content of collagen fibers was lower than that in the other groups. This result indicated that collagenase in Col-M@AuNCs/Dox effectively degraded collagen fibers in the ECM of tumor tissue.

**Fig. 6:**
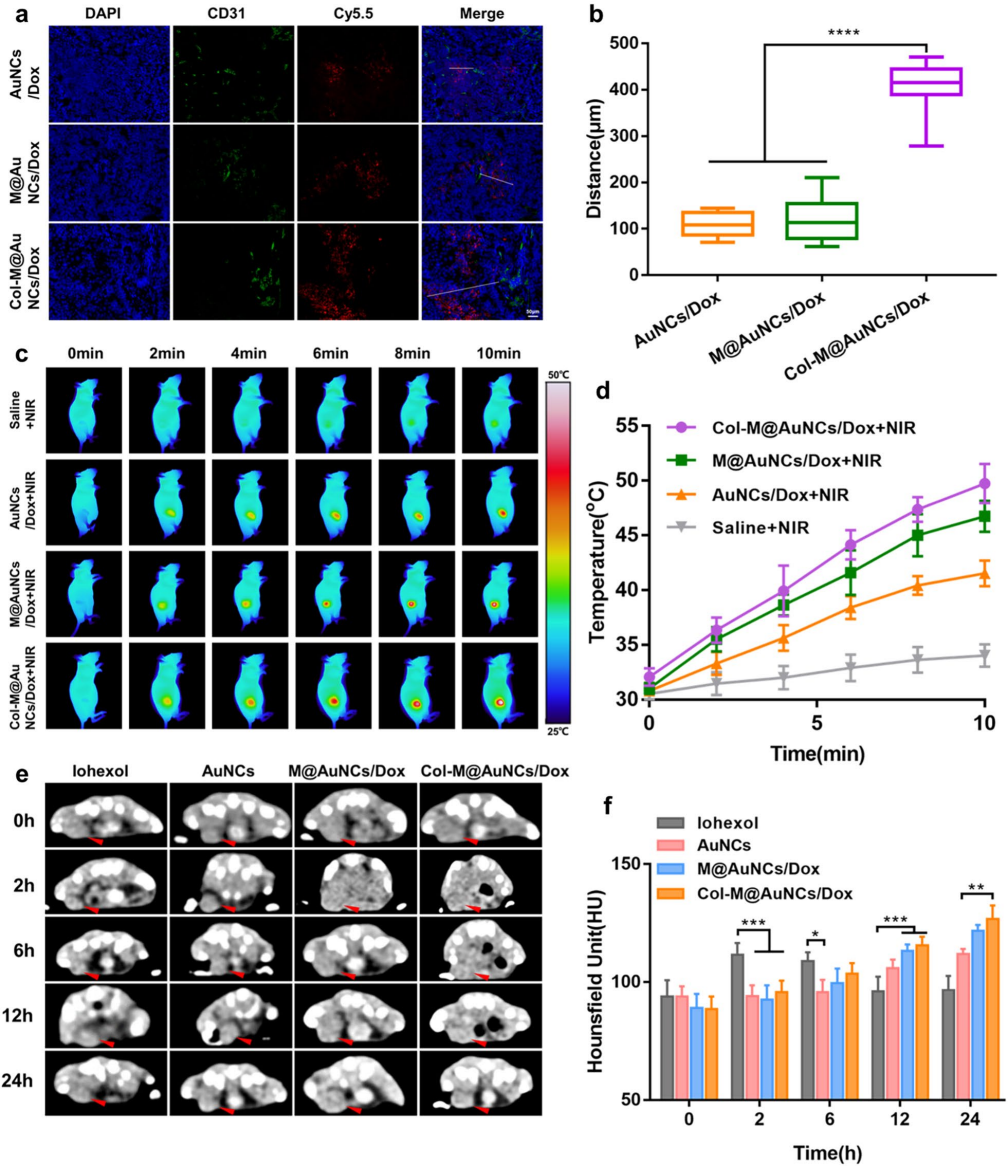
In vivo intratumoral penetration, PTT and PDT efficiency and CT imaging. **a** CLSM images of tumor slices collected from mice receiving AuNCs/Dox, M@AuNCs/Dox and Col-M@AuNCs/Dox. The blue, green and red signals were from the fluorescence of DAPI-stained nuclei, anti-CD31-stained blood vessels and Cy5.5-labeled preparations, respectively. **b** Quantitative analysis of the distance between the nanosamples and blood vessels. **c** Infrared thermal images of BxPC3 tumor-bearing mice intravenously administered different formulations followed by NIR irradiation and (**d**) temperature change curves of NIR-irradiated tumors. **e** In vivo CT imaging of BxPC3 tumor-bearing mice at 0 h, 2 h, 6 h, 12 h and 24 h after intravenous injection of iohexol, AuNCs/Dox, M@AuNCs/Dox and Col-M@AuNCs/Dox (red arrows indicate the tumor site). **f** Quantitative CT values of the tumors in BxPC3 tumor-bearing mice at different time points

Given that Col-M@AuNCs/Dox effectively degraded ECM collagen, we hypothesized that when Col-M@AuNCs/Dox was intravenously administered to BxPC3 tumor-bearing mice, the degradation of ECM collagen could facilitate the diffusion of Col-M@AuNCs/Dox, driving them to penetrate from the regions close to blood vessels to the distal regions. To prove this hypothesis, we visualized the blood vessels using immunofluorescence staining and observed the colocalization of the blood vessels (green) and the nanomaterial preparation (red) by CLSM. As shown in Fig. 6a, in the AuNCs/Dox and M@AuNCs/Dox groups, the fluorescence of the nanomaterial preparation was mainly located near the blood vessels, with a small amount of diffusion from the blood vessels to the distal region. In contrast, the Col-M@AuNCs/Dox group showed a large amount of red fluorescence away from the blood vessels, indicating that Col-M@AuNCs/Dox were able to penetrate deeper than M@AuNCs/Dox and AuNCs/Dox. To more accurately assess the penetration depth of the preparations, ImageJ was used to analyze the distance between the nanosamples and blood vessels. The quantitative analysis results showed that Col-M@AuNCs/Dox penetrated approximately 3.31 times deeper than M@AuNCs/Dox (Fig. 6b). In conclusion, collagen degradation can effectively promote diffusion in tumors, which shows great potential for improving the efficacy of our nanoplatform in vivo.

### In vivo* phototherapy and CT imaging effect*

To study the PTT effect of different preparations in vivo, an infrared thermal imager was used to take photos of the mice under NIR irradiation (Fig. 6c, d). The temperatures of the mice after treatment with saline + NIR increased only slightly and was far from the temperature needed to kill tumor cells. The temperature in the AuNCs/Dox + NIR group increased by approximately 11 °C, and the highest temperature was 42 °C, which was close to the temperature needed to kill tumor cells. The temperature in the M@AuNCs/Dox group increased by approximately 16 °C, and the highest temperature was approximately 47 °C, which was sufficient for tumor cell killing. Col-M@AuNCs/Dox caused a temperature increase of approximately 18 °C, reaching a temperature of 50 °C, which can effectively kill tumor cells, indicating that the matrix degradation effect of collagenase enhanced permeation and accumulation in the tumor site to achieve a better PTT effect.

ROS production in BxPC3 tumor-bearing mice was detected by DCFH-DA staining to investigate the effect of PDT in vivo. As shown in Additional file 1: Fig. S10, DCF fluorescence could hardly be detected in the saline + NIR group. In the Col-M@AuNCs/Dox + NIR group, obvious DCF fluorescence was observed, indicating that Col-M@AuNCs/Dox exerted a good PDT effect in vivo.

We have studied the CT imaging performance of different preparations in vitro. To test whether the preparations could achieve a CT enhancement effect in vivo, CT scans were performed on mice injected with different preparations, and the obtained images and quantitative results are shown in Fig. 6e, f. After injection of iohexol, the tumor site showed rapid CT enhancement within 2 h. Over the next 6, 12, and 24 h, this enhancement at the tumor site gradually subsided. The tumor site was slightly enhanced from 12 to 24 h in the AuNCs group. The enhancement effect in the M@AuNCs/Dox group at the tumor site was better than that in the AuNCs group, which may have been due to the targeted accumulation of the nanosystem mediated by the membrane coating. The enhancement effect in the Col-M@AuNCs/Dox group was further superior to that observed in the M@AuNCs/Dox group, indicating that the degradation effect of collagenase enhanced the penetration and accumulation of the nanosystem at the tumor site to achieve the optimal effect. Therefore, Col-M@AuNCs/Dox can effectively accumulate at the tumor site and achieve a CT enhancement effect, showing great potential for providing an effective means of diagnosis and detection.

Collectively, these results suggest that, when intravenously administered in vivo, Col-M@AuNCs/Dox showed enhanced tumor penetration capacity via ECM degradation. Upon external NIR irradiation, Col-M@AuNCs/Dox showed better PTT, PDT and CT imaging effects than M@AuNCs/Dox and AuNCs/Dox.

### In vivo* antitumor effect and biosafety*

**Fig. 7:**
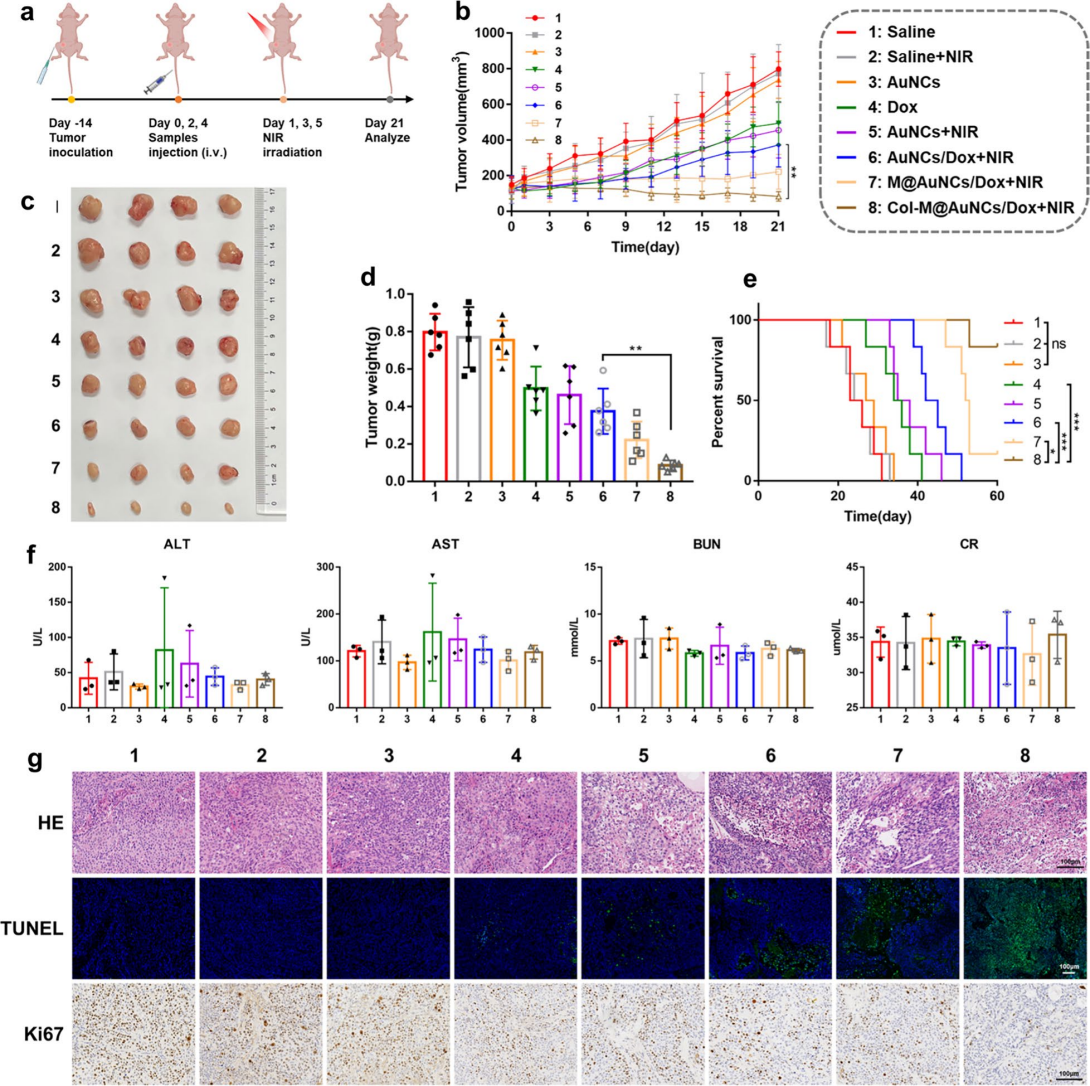
In vivo therapeutic efficacy in BxPC3 tumor-bearing mice. **a** Treatment schedule for the antitumor experiment. **b** Tumor growth curves during different treatments (n = 6). **c** Images of the excised tumors on Day 21. **d** Weights of the excised tumors on Day 21. **e** Survival rate of BxPC3 tumor-bearing mice after receiving different treatments in 60 days (n = 6). **f** Serum ALT, AST, BUN and CR levels in tumor-bearing mice after intervention (n = 3). **g** H&E, TUNEL and Ki67 staining of tumors after mice received different treatments

Due to the enhanced in vitro cytotoxicity, collagen degradation effect and in-tumor penetration ability of the prepared nanoplatform, we further explored the therapeutic effect of the different preparations in BxPC3 tumor-bearing mice. The treatment schedule is shown in Fig. 7a. The tumor growth curves in Fig. 7b showed that saline + NIR and AuNCs treatment had almost no inhibitory effect on tumor growth, which was similar to the saline treatment. Tumor growth was slightly inhibited in the Dox group compared with the saline group. When PTT and PDT effects were induced by NIR irradiation, tumor growth was more significantly inhibited, and the AuNCs/Dox + NIR group showed more effective tumor inhibition than the AuNCs + NIR group, indicating that the combined effect of phototherapy and chemotherapy could inhibit tumor growth more effectively. The M@AuNCs/Dox + NIR group exhibited further inhibited tumor growth, suggesting that the targeting effect of the cell membrane can enhance the in vivo therapeutic effect of the nanoplatform. Finally, the Col-M@AuNCs/Dox + NIR group exhibited the highest extent of tumor growth inhibition among the groups. The spaghetti plot shown in Additional file 1: Fig. S11 showed a similar trend. Figure 7c, d displayed the tumor images and tumor weights after their dissection from the mice, demonstrating that Col-M@AuNCs/Dox + NIR had the strongest antitumor effect. Hematoxylin and eosin (H&E) staining of tumor sections showed that the Col-M@AuNCs/Dox + NIR group had the highest degree of cell necrosis (Fig. 7g). The TdT-mediated dUTP Nick-End Labeling (TUNEL) results consistently showed that the Col-M@AuNCs/Dox + NIR group had the most apoptotic bodies (green) (Fig. 7g). In the Ki67 experiment, the number of Ki67-positive cells in the Col-M@AuNCs/Dox + NIR group was the lowest (brown‒yellow), indicating that Col-M@AuNCs/Dox + NIR could effectively inhibit the proliferation of tumor cells (Fig. 7g). These results were further confirmed by semiquantitative analysis of TUNEL and Ki67 staining images. The Col-M@AuNCs/Dox + NIR group exhibited the greatest proportion of apoptosis and the least proliferative activity among the treatment groups (Additional file 1: Fig. S12). Survival analysis showed that Col-M@AuNCs/Dox + NIR treatment led to significantly longer survival of mice than the other types of treatment (Fig. 7e). Together, these results convincingly proved that Col-M@AuNCs/Dox can effectively target the tumor site and penetrate tumor tissue to exert a powerful and effective antitumor effect under NIR irradiation through combined phototherapy and chemotherapy.

Hemolytic toxicity was studied to examine the safety of Col-M@AuNCs/Dox (Additional file 1: Fig. S13) and RBCs treated with Col-M@AuNCs/Dox showed almost no hemolysis, which was similar to those treated with PBS. In contrast, the RBCs treated with Triton X-100 showed complete hemolysis, demonstrating that Col-M@AuNCs/Dox had good blood safety. In addition, the blood of mice 24 h after injection of Col-M@AuNCs/Dox was collected for hematological analysis and serum biochemical test. As shown in Additional file 1: Table 1 and Fig. S14, compared with the saline group, the Col-M@AuNCs/Dox treated group showed no significant abnormalities in hematological indexes or serum biochemical examination parameters (including ALT, AST, BUN and CR). These results indicated that Col-M@AuNCs/Dox had no obvious short-term toxicological effect postinjection. Finally, at the end of the treatment, body weight, blood biochemical examination results, and H&E staining results obtained from major organs of the mice were analyzed to further evaluate the long-term safety. The results showed that except for the mice in the Dox group with slight weight loss and slightly impaired liver function (ALT and AST), the mice in the other treatment groups showed no significant weight loss (Additional file 1: Fig. S15), no significant abnormalities in liver or kidney function (Fig. 7f), and no histological damage to the major organs (Additional file 1: Fig. S16), indicating that Col-M@AuNCs/Dox have good biosafety in vivo.

Additionally, we conducted animal experiments with free cell membrane treatment to explore whether the free cell membrane has tumorigenic effects. The tumor growth curve in Additional file 1: Fig. S17a indicated that free cell membrane treatment did not promote tumor growth. The final volume and weight of the dissected tumors in the cell membrane-treated group were also similar to those in the saline group (Additional file 1: Fig. S17b, c). In addition, the number of Ki67-positive cells (brown) in the group treated with the cell membrane was similar to those in the saline group (Additional file 1: Fig. S17d, e), indicating that cell membrane treatment did not promote tumor cell proliferation. Overall, the experimental results showed that the free cell membrane has no significant effect on tumor growth and proliferation. Strict procedures are necessary during cell membrane extraction and purification to ensure that no intact cancer cells or nuclear substances remain [47–49]. In our cell membrane extraction procedure, strict and careful manipulation was performed, thus ensuring the purity of the cell membrane we extracted.

Collagenase treatment carries the risk of inducing an immune response when administered intravenously. Considering that immune evasion effects of cell membranes and PEGylation of collagenase contribute to the reduction of immune responses [47, 50, 51], we briefly evaluated proinflammatory responses in vivo. The in vivo inflammatory response was evaluated in the serum of BALB/c mice at 24 h after intravenous injection with saline, collagenase and Col-M@AuNCs/Dox by enzyme-linked immunosorbent assay (ELISA). As shown in Additional file 1: Fig. S18, collagenase led to slightly higher IL-6 contents in serum than the Col-M@AuNCs/Dox treatment. The results revealed that the covalent conjugation of PEG to collagenase and the immune escape effect of the cell membrane in our nanosystem may contribute to decreasing the immune inflammatory response induced by collagenase, which further revealed the good biosafety of Col-M@AuNCs/Dox.

Inspired by the potent tumor-killing effect of Col-M@AuNCs/Dox in vivo, the efficacies of Col-M@AuNCs/Dox and nab-paclitaxel (a nanomedicine used in clinical practice) were compared and further investigated. The results in Additional file 1: Fig. S19 showed that Col-M@AuNCs/Dox have a more potent antitumor effect than the clinically used nab-paclitaxel. In 2013, nab-paclitaxel in combination with gemcitabine was approved by the FDA as a first-line treatment for PDAC [52]. Although combination therapy improved survival to some extent, the corresponding monotherapy did not show a statistical improvement over standard therapy [4, 53]. Consistent with our experimental results, nab-paclitaxel treatment alone had a limited inhibitory effect on tumor growth compared with Col-M@AuNCs/Dox. Nab-paclitaxel is a 130 nm albumin-bound formulation of paclitaxel particles, and its intratumoral accumulation and efficacy depend on the EPR effect of the tumor [54, 55]. However, PDAC is a tumor rich in dense stroma, and its low permeability poses a challenge to EPR effect-based therapeutic agents. Therefore, we concluded that the limited efficacy of nab-paclitaxel may be due to the insufficient EPR effect in stroma-rich PDAC. In contrast, since Col-M@AuNCs/Dox could accumulate and permeate into PDAC tumors due to their active targeting ability and ability to degrade the ECM, multidirectional combination therapy can be achieved to exert a potent tumor killing effect. Therefore, the above results and analysis indicated that Col-M@AuNCs/Dox have advantages over clinically used nanomedicine to overcome the therapeutic barriers of PDAC.

## Conclusions

In this work, we generated a versatile nanoplatform Col-M@AuNCs/Dox that enhances the treatment of PDAC by integrating active targeting, immune evasion, deep penetration, PDT/PTT/chemotherapy and CT imaging. More specifically, Col-M@AuNCs/Dox possessed the following advantages, which are convincingly supported by our experimental data. (1) Collagenase-functionalized biomimetic Col-M@AuNCs/Dox were prepared under simple conditions with easy steps and displayed appropriate stability and biosafety. (2) Upon intravenous injection, Col-M@AuNCs/Dox can prevent nonspecific immune uptake, actively target PDAC tumor tissues, effectively degrade dense ECM, and penetrate deeply into the tumor parenchyma. (3) In terms of the tumor-killing effect, under 808 nm NIR irradiation, the chemotherapeutic drugs in Col-M@AuNCs/Dox were released in a controlled manner. Additionally, Col-M@AuNCs/Dox exerted combined PTT and PDT killing effects on PDAC, showing the strongest tumor inhibitory effect in BxPC3 tumor-bearing mice. (4) Considering the imaging effect, Col-M@AuNCs/Dox can achieve an effective CT enhancement effect at the tumor site. (5) This effective ECM regulation strategy may also apply to other stroma-rich tumors to solve the problem of poor drug penetration in stroma-rich tumors. In conclusion, the use of Col-M@AuNCs/Dox provided a simple and effective strategy for the generation of a multifunctional nanoplatform for the thorough elimination of PDAC by simultaneously integrating the properties of deep tumor penetration, enhanced tumor-killing effects and effective imaging capability, providing an efficacious approach to the dilemma of treating PDAC.

## Methods

### Materials

Ethylene glycol (EG), poly(vinyl pyrrolidone) (PVP), silver trifluoroacetate (CF_3_COOAg), doxorubicin hydrochloride (Dox), 2-iminothiolane (Traut’s reagent), MTT and iohexol were obtained from Aladdin Co., Ltd. (Shanghai, China). Sodium hydrosulfide hydrate (NaHS) was purchased from Saen Chemical Technology Co., Ltd (Shanghai, China). Chloroauric acid (HAuCl_4_) was obtained from Shanghai Titan Scientific Co., Ltd. (Shanghai, China). Membrane and Cytosol Protein Extraction Kit, DCFH-DA, 4′6-diamidino-2-phenylindole (DAPI) and Annexin V-FITC/PI Apoptosis Detection Kit were purchased from Beyotime (China). Collagenase (Type I) was purchased from Sigma-Aldrich (St. Louis, MO, USA). Rattailtendon collagen type I and Calcein-AM/PI kit were purchased from Solarbio (Beijing, China). DSPE-PEG_2k_-MAL was purchased from ShangHai PonsureBio Tech. Inc. (Shanghai, China). SH-PEG-Cy5.5 was purchased from ShangHai ToYongBio Tech.Inc. (Shanghai, China). N-cadherin antibody, anti-CD47 antibody, EpCAM antibody, ATP1A1 antibody, collagen type I antibody and anti-CD31 antibody were purchased from Proteintech (Wuhan, China). Nab-paclitaxel was purchased from Qilu pharmaceutical (Hainan, China). IL-6, IL-1β and TNF-α ELISA kits were obtained from Jiangsu Meimian Industrial Co., Ltd. (Jiangsu, China).

### Cell lines and animals

Human pancreatic cancer cell line BxPC3, murine hepatoma cell line H22, murine melanoma cell line B16, murine breast cancer cell line 4T1 and human THP-1 cell line were purchased from Procell (Wuhan, China). BxPC3, H22, B16, 4T1 and THP-1 cells were cultured in RPMI 1640 medium containing 10% FBS at 37 °C in a 5% CO_2_ humidified incubator. The THP-1 cells were differentiated into a macrophage-like phenotype by incubating in 100 ng/mL phorbol myristate acetatefor 48 h.

Female BALB/c nude mice and BALB/c mice (4–6 weeks) were obtained from the Shanghai Silaike Laboratory Animal Limited Liability Company. All animal experiments were performed following the care and use guidelines of the National Institutes of Health (NIH, USA) and approved by the Animal Experiment Committee of Zhejiang University. To establish a BxPC3 tumor-bearing mice model, female BALB/c nude mice were subcutaneously injected with 2.0 ×10^6^ cells in the right thigh and kept until the tumor volume was appropriate for experiments. The tumor volume was calculated as V = a^2^ ×b/2, where ‘a’ represents the minimum length (mm) of the tumor, and ‘b’ represents the maximum length (mm) of the tumor.

### Preparation of Au nanocages (AuNCs) and Dox loaded Au nanocages (AuNCs/Dox)

AuNCS were synthesized by using a galvanic replacement reaction between Ag nanocubes (AgNCs) and HAuCl_4_ as previously reported [56, 57]. To prepare AgNCs, EG (5 mL) was heated to 150 °C under stirring. NaHS solution (3 mM), HCl solution (3 mM), PVP solution (20 mg/mL) and CF_3_COOAg solution (282 mM) were successively added into EG to react for 15 min. Then the AgNCs were collected by centrifugation and washed with acetone once and deionized (DI) water. To obtain AuNCs, the AgNCs were added into PVP aqueous solution heated to 90 °C, then HAuCl_4_ aqueous solution was injected into the reaction solution at the rate of 0.75 mL/min until the solution had an LSPR peak at 800 nm monitored by UV‒Vis spectroscopy (UV-2600, SHIMADZU, Japan). The AuNCs were collected by centrifugation, washed with DI water and then dispersed in DI water for later experiments. To prepare AuNCs/Dox, Dox (1.5 mg) was dissolved in 10 mL of AuNCs solution and stirred under dark conditions for 48 h. The final mixture was centrifuged and washed with PBS to remove the unloaded Dox. The supernatant was collected and the content of unloaded drugs in the supernatant was determined by a fluorescence spectrophotometer (FP 6500, JASCO, Japan). Encapsulation efficiency (EE) and drug loading capacity (DLC) were calculated according to the following equations

$${\text{EE }}(\% ) = \left( {{\text{weight of loaded drug}}/{\text{weight of devoted drug}}} \right) \times {1}00\%$$

$${\text{DLC}}(\% ) = \left( {{\text{weight of loaded drug }}/{\text{total weight of drug carrier}}} \right) \, \times {1}00\%$$

### Extraction of BxPC3 cell membranes and preparation of cell membranes coated AuNCs/Dox (M@AuNCs/Dox)

The extraction of the BxPC3 cell membranes was performed based on the manufacturer’s directions of the membrane protein extraction kit (Beyotime, China). In brief, the cultured BxPC3 cells were collected and suspended with membrane protein extraction reagent A, and placed in ice bath for 15 min. Then the cells were fully lysed by freezing at liquid nitrogen and thawing at room temperature for four times. Centrifugation was performed at 700 g at 4 °C for 10 min to precipitate unbroken cells and nuclei. The supernatant was collected carefully. Then the cell membranes were precipitated by centrifugation at 14000 g at 4 °C for 30 min. Discard the supernatant and collect the precipitation as the extracted BxPC3 cell membranes. To prepare M@AuNCs/Dox, AuNCs/Dox were mixed with BxPC3 cell membranes under sonication and then extruded through 200 nm polycarbonate membranes (LiposoFast, Avestin, Canada). M@AuNCs/Dox were collected by centrifugation and washed with DI water.

### Preparation of Col-M@AuNCs/Dox

To obtain Col-M@AuNCs/Dox, Collagenase-functionalized DSPE-PEG-MAL (DSPE-PEG-Col) was first synthesized. Firstly, DSPE-PEG-Col was synthesized as reported previously with minor modifications [58]. Traut’s reagent (8 mg/mL) and collagenase (5 mg/mL) were mixed and shaken at room temperature for 1 h to obtain thiolated collagenase (SH-Col). The excess Traut’s reagent was removed by dialysis (MWCO = 3500 Da). DSPE-PEG-MAL (10 mg/mL) and SH-Col (5 mg/mL) were mixed and shocked at room temperature to obtain DSPE-PEG-Col, and excess DSPE-PEG-MAL was removed by dialysis. The synthesis of DSPE-PEG-Col was detected by ^1^H NMR (BRUKER AVIII500M, Switzerland). Then, DSPE-PEG-Col was added into M@AuNCs/Dox and incubated at room temperature for 1 h to allow DSPE-PEG-Col inserting into the cell membrane by lipid fusion. The un-linked DSPE-PEG-Col was removed by dialysis. Col-M@AuNCs/Dox were collected by centrifugation and washed with DI water.

### Characterization

The particle size and zeta potential were measured by particle analyzer (Litesizer500, Anton-Paar, Austria). The morphologies of AgNCs, AuNCs and M@AuNCs/Dox were observed by TEM (JEM-1400flash, JEOL, Japan). For colloidal stability analysis, AuNCs, AuNCs/Dox, M@AuNCs/Dox and Col-M@AuNCs/Dox were dispersed in PBS (pH 7.4) supplemented with 10% FBS at room temperature. The mean particle size and zeta potential of Col-M@AuNCs/Dox were recorded in 7 days. The UV‒Vis absorption spectra of AgNCs and AuNCs were characterized by UV‒Vis spectrophotometer (UV-2600, SHIMADZU, Japan). The concentration of Au ions was measured by ICP‒MS (PerkinElmer NexION 300X, USA). The release of Dox in AuNCs/Dox, M@AuNCs/Dox, M@AuNCs/Dox + NIR and Col-M@AuNCs/Dox + NIR was determined by dynamic dialysis method (MWCO = 3500 Da) in PBS. At predetermined time points, 2 mL of the release media was collected and 2 mL of fresh medium was added, and the contents of Dox released were determined by a fluorescence spectrophotometer (FP 6500, JASCO, Japan). SDS‒PAGE assay was applied to characterize the external proteins of M@AuNCs/Dox, and the expressions of CD47, EpCAM, N-cadherin and ATP1A1 proteins were detected by Western blotting. The binding rate of collagenase on the nanosystem was measured by the Enhanced BCA Protein Assay Kit (Beyotime, China). The unbound collagenase was collected by ultrafiltration, and the content of unbound collagenase was measured by BCA protein assay, and then the binding rate of collagenase was calculated.

### Collagenase activity

The remaining enzymatic activity of collagenase was preliminarily investigated by gelatin. In brief, gelatin (30 mg/mL) solution was co-cultured with free collagenase, DSPE-PEG-Col, M@AuNCs/Dox and Col-M@AuNCs/Dox at 37 °C. After 24 h, each sample was then stored at 4 °C for 30 min before being imaged by a digital camera. Besides, the collagenase activity was further quantified by Collagenase Activity Colorimetric Assay Kit (Sigma-Aldrich, St. Louis, MO, USA).

### In vitro* PTT, PDT and CT imaging effects*

The photothermal profiles in PBS were recorded by digital thermometer (DT1311, LiHuaJin, China) upon irradiation of an 808 nm laser (LSR808NL, LASEVER INC, China) at 1 W/cm^2^ for 10 min, and an infrared thermal camera (Zcec-140015f, Nippon Avionics, Japan) was used to record the thermographic images. Besides, to evaluate the photothermal stability, Col-M@AuNCs/Dox dispersion was irradiated and the temperature changes during five heating–cooling cycles were recorded.

DPBF was used to detect the generation of singlet oxygen (^1^O_2_) in aqueous solution. Col-M@AuNCs/Dox solution was mixed with DPBF dissolved in DMSO (20 μL, 0.5 mg/mL). The absorbance of DPBF at 410 nm was measured by UV‒Vis spectrophotometer after being irradiated by 808 nm laser at 1 W/cm^2^. DCFH-DA fluorescent probe was used to detect the ability of Col-M@AuNCs/Dox to produce ROS in cells. BxPC3 cells were seeded in 24-well plates and incubated with PBS, AuNCs and Col-M@AuNCs/Dox (50 μg/mL) for 4 h. Then all cells were co-incubated with DCFH-DA (10 μM) for 30 min, followed by 808 nm laser irradiation (1 W/cm^2^, 10 min) or not. The production of ROS was analyzed by CLSM (A1R, Nikon, Japan) and FCM (ACEA NovoCyteTM, ACEA Biosciences, USA).

Various Au concentrations of AuNCs/Dox, M@AuNCs/Dox and Col-M@AuNCs/Dox (0 mM, 1 mM, 3 mM, 4 mM, 5 mM) and various I concentrations of iohexol (0 mM, 1 mM, 3 mM, 4 mM, 5 mM) were prepared in 2 mL Eppendorf tubes and scanned by a CT scanner (SOMATOM Force, SIEMENS) with scanning parameters of 50 mAs, 70 kV, scanning time of 0.28 s and layer thickness of 0.6 mm. The RadiAnt DICOM Viewer software was used to measure Hounsfield units (HU) for each region of interest (ROI). The linear relationship was obtained by fitting plots of the HU and Au concentration (mM).

### In vitro* Collagen degradation behavior in ECM-mimicking gel*

Collagen was mixed with PBS containing FITC (10 μg/mL) in an ice bath, then the solution was moved to a 24-well plate and left at room temperature for 20 min to promote gelation. Once the gel was formed, wash the gel gently until negligible fluorescence was observed in the supernatant. Then 200 μL of PBS, M@AuNCs/Dox, Col-M@AuNCs/Dox (50 μg/mL) and collagenase were added to the gel surface, respectively, and the fluorescence in the supernatant was detected after incubation at 37 °C for 24 h.

To prepare capillaries containing collagen gel, the collagen, DI H_2_O, NaOH and 10X PBS were successively mixed in ice bath and the mixture was added into the capillary (0.5 mm diameter). After the gel was formed in capillary tube, 10 μL of Dox, M@AuNCs/Dox and Col-M@AuNCs/Dox (50 μg/mL) were injected into the head of the capillary and co-cultured for 24 h. The fluorescence intensity of the head, middle, and tail of capillary was observed under CLSM.

### Cell uptake, macrophage escape and Dox intracellular release study

To investigate the cell uptake of different nanopreparations, the BxPC3 cells were plated into 24-well plates and cultured with AuNCs, AuNCs/Dox, M@AuNCs/Dox and Col-M@AuNCs/Dox (all labeled with Cy5.5) for 4 h or 12 h. Finally, CLSM was used for observation and analysis. For ICP‒MS analysis, the cells were washed with PBS three times and then dissolved in aqua regia (containing HNO_3_: HCl = 1:3, v/v). The intracellular Au contents were determined by ICP‒MS.

To investigate the macrophage escape ability of the nanoplatform, the macrophage-like phenotype THP-1 cells were coincubated with PBS, AuNCs/Dox and M@AuNCs/Dox for 4 h. Finally, the cells were collected for FCM analysis.

To further demonstrate the intracellular Dox release upon 808 nm laser irradiation, BxPC3 cells were plated into 24-well plates and incubated with Dox, AuNCs/Dox, M@AuNCs/Dox and Col-M@AuNCs/Dox and exposed to irradiation (808 nm, 1 W/cm^2^, 10 min) or not. Finally, the cells were stained with DAPI and observed under CLSM.

### *Cytotoxicity of Col-M@AuNCs/Dox *in vitro

For MTT assay, the BxPC3 cells were plated in 96-well plates and incubated with PBS, Dox, AuNCs, AuNCs/Dox, M@AuNCs/Dox and Col-M@AuNCs/Dox (50 μg/mL) for 12 h, followed by laser irradiation (808 nm, 1 W/cm^2^, 10 min) or dark treatment. Afterwards, cells were further incubated for 12 h. Then 20ul MTT solution (5 mg/mL) was added into each well and incubated for 4 h. The medium was carefully removed and 150 µL of DMSO was added in each well to dissolve the formazan. The OD value was measured using a Microplate reader (ID5, Molecular Devices, USA) at a wavelength of 570 nm.

For live/dead assay, the BxPC3 cells were plated in 12-well plates and incubated with AuNCs, AuNCs/Dox, M@AuNCs/Dox and Col-M@AuNCs/Dox (50 μg/mL) for 12 h, followed by laser irradiation (808 nm, 1 W/cm^2^, 10 min) or dark treatment. Afterwards, cells were further incubated for 12 h followed by PBS washing, collection and Calcein-AM/PI staining. Finally, the cells were observed under a fluorescent inverted microscope (Axio Observer A1, Germany).

For apoptosis analysis, the BxPC3 cells were plated in 24-well and incubated with PBS, Dox, AuNCs, AuNCs/Dox, M@AuNCs/Dox and Col-M@AuNCs/Dox (50 μg/mL) for 12 h, followed by laser irradiation (808 nm, 1 W/cm^2^, 10 min) or dark treatment. Afterwards, cells were further incubated with fresh medium for 12 h followed by trypsin (without EDTA) digestion, collection, Annexin V-FITC/PI staining and FCM analysis.

### Penetration and toxicity in 3D BxPC3 multicellular tumor spheroids (MTSs)

The BxPC3 MTSs were established as follows. Briefly, agarose was coated on the bottom of 96-well plates by adding 100 μL of 1.5% hot agarose solution in each well and then incubating at 37 °C for 24 h. The BxPC3 cells were plated in the agarose-coated plates at a density of 1 ×10^3^ cells/well. After 7 days of incubation, the BxPC3 MTSs formed and those with a diameter around 200 μm were selected for further experiments.

To investigate the penetration of different nanoplatforms, the prepared BxPC3 MTSs were transferred to a confocal dish with 1 mL fresh medium containing free Dox, AuNCs/Dox, M@AuNCs/Dox and Col-M@AuNCs/Dox (50 μg/mL). After 24 h of incubation, all MTSs were washed with PBS and observed using CLSM in Z-stacks images.

For the MTSs growth inhibition study, the prepared BxPC3 MTSs were co-incubated free Dox, AuNCs, AuNCs/Dox, M@AuNCs/Dox and Col-M@AuNCs/Dox (50 μg/mL). After 24 h of incubation, MTSs were irradiated upon 808 nm laser or not (1 W/cm^2^, 10 min). All MTSs were cultured for 7 days, and the growth of the MTSs was observed and photographed by an optical microscope (Axio Observer A1, Germany).

### In vivo* biodistribution*

When tumors reached approximately 300 mm^3^, BxPC3 tumor-bearing mice were randomly divided into three groups (n = 3), intravenously receiving Cy5.5-labeled AuNCs/Dox, M@AuNCs/Dox and Col-M@AuNCs/Dox (10 mg/kg). For in vivo imaging, mice were imaged with the in vivo fluorescence imaging system (IVIS Spectrum, Caliper, USA) at predetermined time points (2, 6, 12 and 24 h). For ex vivo imaging, mice were sacrificed 24 h post injection and the tumors and major organs were collected and imaged. In addition, the tumors and major organs were collected and completely digested with aqua regia. Au contents were determined by ICP‒MS and expressed as percent of the injected Au dose per gram of tissue.

### In vivo* collagen degradation effect and tumor penetration*

Masson’s trichrome and Immunofluorescence staining was used to investigate the degradation of collagen within the tumor. BxPC3 tumor-bearing mice were sacrificed at 24 h post injection of saline, AuNCs/Dox, M@AuNCs/Dox and Col-M@AuNCs/Dox (10 mg/kg). The collected tumors were prepared into paraffin sections. These sections were stained with a Masson’s trichrome kit to detect collagen fiber in tumor tissues and imaged under microscope. In addition, sections were incubated with primary antibodies to collagen I and secondary antibody according to the kit’s instructions.

To evaluate in vivo tumor penetration capacity of Col-M@AuNCs/Dox, BxPC3 tumor-bearing mice were intravenously injected AuNCs/Dox, M@AuNCs/Dox and Col-M@AuNCs/Dox (10 mg/kg). The mice were sacrificed 24 h later, and the tumors were collected, frozen and cutted into slices. The tumor sections were subjected to DAPI and CD31 staining, and the in vivo tumor penetration was evaluated by CLSM analysis.

### In vivo* PTT, PDT and CT imaging effects*

BxPC3 tumor-bearing mice were intravenously injected with saline, AuNCs/Dox, M@AuNCs/Dox and Col-M@AuNCs/Dox (10 mg/kg). At 24 h after injection, the mice were irradiated with 808 nm laser at 1 W/cm^2^ for 10 min. The mice were photographed with infrared thermal imaging camera, and the photothermal temperatures at different time points were recorded.

The ROS generation in vivo was detected using DCFH-DA as a probe. Briefly, BxPC3 tumor-bearing mice were intravenously injected with saline and Col-M@AuNCs/Dox (10 mg/kg). After 24 h, the mice were intratumorally injected with DCFH-DA and exposed to 808 nm laser (1 W/cm^2^, 10 min) or not. Finally, the mice were sacrificed and tumors were collected, rinsed, frozen, cutted into slices, and stained with DAPI before CLSM examination.

BxPC3 tumor-bearing mice were intravenously injected with 200 μL of AuNCs/Dox, M@AuNCs/Dox, Col-M@AuNCs/Dox and iohexol through tail vein. CT scans were performed before and 2, 6, 12 and 24 h after injection with the same scan parameters as in vitro experiment. HU of each ROI at the tumor site was measured using RadiAnt DICOM Viewer software.

### In vivo* antitumor effect and biosafety*

When the tumor volume reached about 100–200 mm^3^, BxPC3 tumor-bearing mice were randomly divided into eight groups (n = 6) and treated with saline, saline + NIR, AuNCs, Dox, AuNCs + NIR, AuNCs/Dox + NIR, M@AuNCs/Dox + NIR and Col-M@AuNCs/Dox (10 mg/kg) + NIR respectively. At 24 h post-injection, the mice in “ + NIR” groups were irradiated by an 808 nm laser for 5 min (1 W/cm^2^). The injection was performed on days 0, 2, and 4. The NIR irradiation was performed on days 1, 3 and 5. Body weight and tumor size were recorded every two days. On day 21, all mice were sacrificed and the blood serum was collected for hepatorenal function analysis. The tumors and major organs were collected for H&E staining. TUNEL assays of tumor sections in all groups were conducted to detect the tumor apoptosis, wherein nuclei stained with green were defined as TUNEL-positive nuclei. The tumor samples were also stained with Ki67. Image J software was used for semi-quantitative analysis of TUNEL and Ki67 staining images of tumor tissues. For survival analysis, BxPC3 tumor-bearing mice were treated with the above treatment. The survival of mice in each group was recorded within 60 days. For hemolysis assay, red blood cells (RBCs) were incubated with PBS (negative control), Col-M@AuNCs/Dox and Triton X -100 (positive control) at 37 °C in PBS for 1 h and the centrifuge tube was photographed and observed. For short-term safety evaluation, the blood was collected from BxPC-3 tumor-bearing mice 24 h after intravenous administration of saline and Col-M@AuNCs/Dox. The collected whole blood was used for hematological analysis, and serum was separated from the blood for ALT, AST, BUN and CR examination.

For the free cell membrane animal study, the BxPC3 tumor-bearing mice were treated with saline and free cell membrane respectively and the tumor size was recorded. On day 21, all mice were sacrificed, and the tumors were dissected, photographed and weighed. In addition, the tumors were collected for Ki67 immunohistochemical staining.

For the immune response study of collagenase, BALB/c mice were randomly divided into three groups and intravenously injected with saline, collagenase and Col-M@AuNCs/Dox, respectively. After 24 h of treatment, blood samples were collected, serum was separated, and IL-1β, IL-6 and TNF-α activities were detected by ELISA.

Regarding the comparison of efficacy with clinical nanomedicine nab-paclitaxel, the procedure is similar to the experimental procedure described above. Briefly, mice were treated with saline, nab-paclitaxel, and Col-M@AuNCs/Dox + NIR, and tumor growth curves were recorded. In addition, the tumors were collected for H&E, TUNEL and Ki67 staining after treatment.

### Statistical analysis

Statistical analysis was carried out with GraphPad Prism software. Data are presented as mean ± SD. Comparison between groups was performed using one-way analysis of variance (ANOVA) and Student’s t test, and P < 0.05 was considered significant. *p < 0.05, **p < 0.01, ***p < 0.001. ****p < 0.0001.

## Supplementary Information

**Additional file 1: Fig. S1.** 1H NMR of DSPE-PEG-Col. **Fig. S2.** Pictures of gelatin solutions cocultured with different preparations. **Fig. S3.** Stability of AuNCs, AuNCs/Dox, M@AuNCs/Dox and Col-M@AuNCs/Dox. **Fig. S4.** Protein profiles of BxPC3 cell, BxPC3 cell membrane and M@AuNCs by SDS-PAGE. **Fig. S5**. Flow cytometry analysis of macrophage-like phenotype THP-1 cells after incubation with M@AuNCs/Dox or AuNCs/Dox. **Fig. S6**. Temperature elevation curve of PBS and Col-M@AuNCs/Dox at different NIR laser power irradiation and different concentration. **Fig. S7**. Flow cytometry analysis of ROS generation in BxPC3 cells. **Fig. S8**. CLSM observation of Dox release profiles in BxPC3 cells with or without NIR irradiation. **Fig. S9**. Cell viability of BxPC3 cells treated with AuNCs and collagenase at different concentration. **Fig. S10**. ROS detection in BxPC3 tumor slices observed by CLSM. **Fig. S11**. Tumor spaghetti curves of BxPC3 tumor bearing mice with different treatment. **Fig. S12.** Semi-quantitative analysis of (**a**) TUNEL and (**b**) Ki67 staining images. **Fig. S13**. Hemolysis analysis. **Table 1.** Hematological analysis of mice post-injection of Col-M@AuNCs/Dox and saline. **Fig. S14.** Blood biochemical test of mice post-injection of Col-M@AuNCs/Dox and saline. **Fig. S15**. Body weights of the mice during treatment. **Fig. S16**. H&E staining of the major organs extracted from BxPC3 tumor bearing mice after the 21 days treatment.** Fig. S17.** **a** Tumor growth curves of mice after receiving saline and cell membrane treatment. **b** Images of the excised tumors on Day 21. **c** Weights of the excised tumors on Day 21. **d** Ki67 staining of tumors after mice receiving saline and cell membrane treatments. **e** Semi-quantitative analysis of the Ki67 staining images. **Fig. S18.** IL-6, IL-1β and TNF-α contents in serum of mice post-injection of saline, Col-M@AuNCs/Dox and collagenase. **Fig. S19. a** Tumor growth curves of mice after receiving saline, nab-paclitaxel and Col-M@AuNCs/Dox+NIR treatment. **b** Images of the excised tumors on Day 21. **c** Weights of the excised tumors on Day 21. **d** H&E, TUNEL and Ki67 staining of tumors after mice receiving different treatments. **e** Semi-quantitative analysis of TUNEL staining images. **f** Semi-quantitative analysis of Ki67 staining images.

## Data Availability

All data generated or analyzed during this study are included in this published article and its supplementary information files.
