# Supplementary material for: Extracellular matrix modulating enzyme functionalized biomimetic Au nanoplatform-mediated enhanced tumor penetration and synergistic antitumor therapy for pancreatic cancer
Source: J Nanobiotechnology. 2022 Dec 10;20:524. doi: 10.1186/s12951-022-01738-6 (PMC9741808; doi:10.1186/s12951-022-01738-6)
Supplement: Supplementary file 1 — Additional file 1: Fig. S1. 1H NMR of DSPE-PEG-Col. Fig. S2. Pictures of gelatin solutions cocultured with different preparations. Fig. S3. Stability of AuNCs, AuNCs/Dox, M@AuNCs/Dox and Col-M@AuNCs/Dox. Fig. S4. Protein profiles of BxPC3 cell, BxPC3 cell membrane and M@AuNCs by SDS-PAGE. Fig. S5. Flow cytometry analysis of macrophage-like phenotype THP-1 cells after incubation with M@AuNCs/Dox or AuNCs/Dox. Fig. S6. Temperature elevation curve of PBS and Col-M@AuNCs/Dox at different NIR laser power irradiation and different concentration. Fig. S7. Flow cytometry analysis of ROS generation in BxPC3 cells. Fig. S8. CLSM observation of Dox release profiles in BxPC3 cells with or without NIR irradiation. Fig. S9. Cell viability of BxPC3 cells treated with AuNCs and collagenase at different concentration. Fig. S10. ROS detection in BxPC3 tumor slices observed by CLSM. Fig. S11. Tumor spaghetti curves of BxPC3 tumor bearing mice with different treatment. Fig. S12. Semi-quantitative analysis of (a) TUNEL and (b) Ki67 staining images. Fig. S13. Hemolysis analysis. Table 1. Hematological analysis of mice post-injection of Col-M@AuNCs/Dox and saline. Fig. S14. Blood biochemical test of mice post-injection of Col-M@AuNCs/Dox and saline. Fig. S15. Body weights of the mice during treatment. Fig. S16. H&E staining of the major organs extracted from BxPC3 tumor bearing mice after the 21 days treatment. Fig. S17. a Tumor growth curves of mice after receiving saline and cell membrane treatment. b Images of the excised tumors on Day 21. c Weights of the excised tumors on Day 21. d Ki67 staining of tumors after mice receiving saline and cell membrane treatments. e Semi-quantitative analysis of the Ki67 staining images. Fig. S18. IL-6, IL-1β and TNF-α contents in serum of mice post-injection of saline, Col-M@AuNCs/Dox and collagenase. Fig. S19. a Tumor growth curves of mice after receiving saline, nab-paclitaxel and Col-M@AuNCs/Dox+NIR treatment. b Images of the excised tumors on Day [file 12951_2022_1738_MOESM1_ESM.docx]

**Supporting Information**

**Extracellular Matrix Modulating Enzyme Functionalized Biomimetic Au nanoplatform-Mediated Enhanced Tumor Penetration and Synergistic Antitumor Therapy for Pancreatic Cancer**

Xiao-Yan Yang^1^, Jin-Guo Zhang^1^, Qiao-Mei Zhou^1^, Jie-Ni Yu^1^, Yuan-Fei Lu^1^, Xiao-Jie Wang^1^, Jia-Ping Zhou^1^, Xin-Fa Ding^1^, Yong-Zhong Du^2, #^, Ri-Sheng Yu^1, #^

^1^Department of Radiology, Second Affiliated Hospital, School of Medicine, Zhejiang University, 88 Jiefang Road, Hangzhou, 310009 Zhejiang Province People’s Republic of China

^2^Institute of Pharmaceutics, College of Pharmaceutical Sciences, Zhejiang University, 866 Yuhangtang Road, Hangzhou, 310058 Zhejiang Province People’s Republic of China

^#^ Corresponding author

First corresponding author: Ri-Sheng Yu, Professor, Department of Radiology, Second Affiliated Hospital, School of Medicine, Zhejiang University, 88 Jiefang Road, Hangzhou 310009, Zhejiang Province, People’s Republic of China. Tel: +86-571-87783925. E-mail: risheng-yu@zju.edu.cn

Second corresponding author: Yong-Zhong Du, Professor, Institute of Pharmaceutics, College of Pharmaceutical Sciences, Zhejiang University, 866 Yuhangtang Road, Hangzhou 310058, Zhejiang Province, People’s Republic of China. Tel: +86-571-88208435. E-mail: duyongzhong@zju.edu.cn


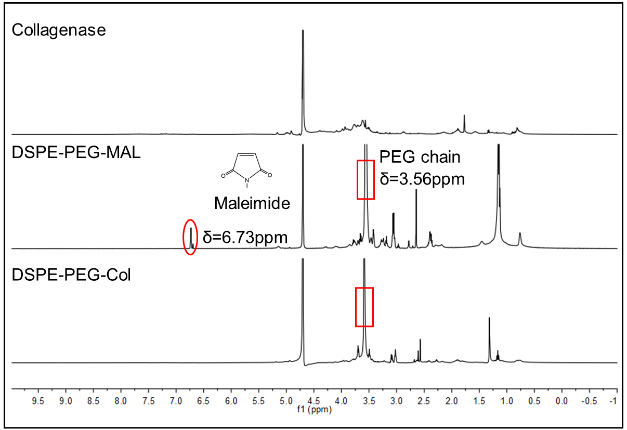


**Fig. S1.** ^1^H NMR of DSPE-PEG-Col.


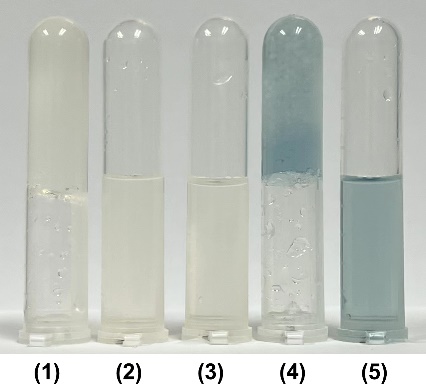


**Fig. S2.** Gelatin solutions were cocultured with different preparations at 37 °C and then stored at 4 °C for 30 min: (1) Pure gelatin; (2) Collagenase; (3) DSPE-PEG-Col; (4) M@AuNCs/Dox; (5) Col-M@AuNCs/Dox.


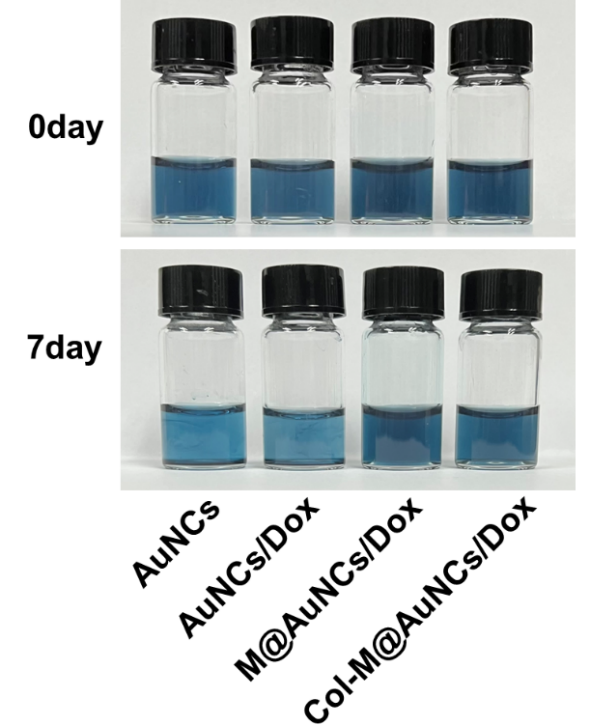


**Fig. S3.** Pictures of AuNCs, AuNCs/Dox, M@AuNCs/Dox and Col-M@AuNCs/Dox in PBS containing 10% FBS placed at room temperature for a week.


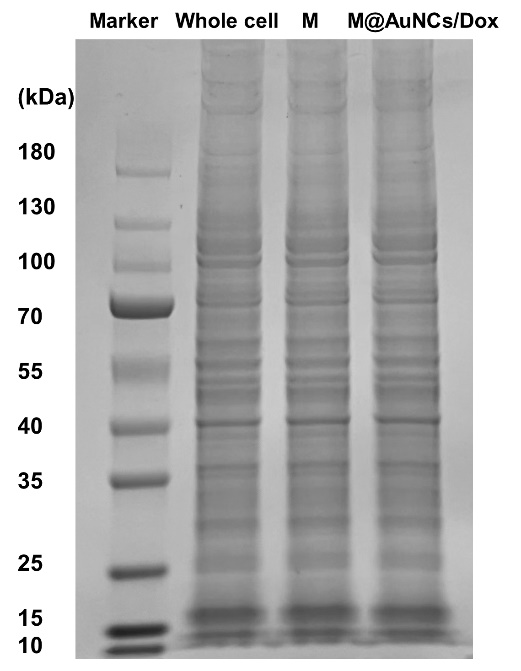


**Fig. S4.** Protein profiles of BxPC3 cell, BxPC3 cell membrane and M@AuNCs by SDS-PAGE.


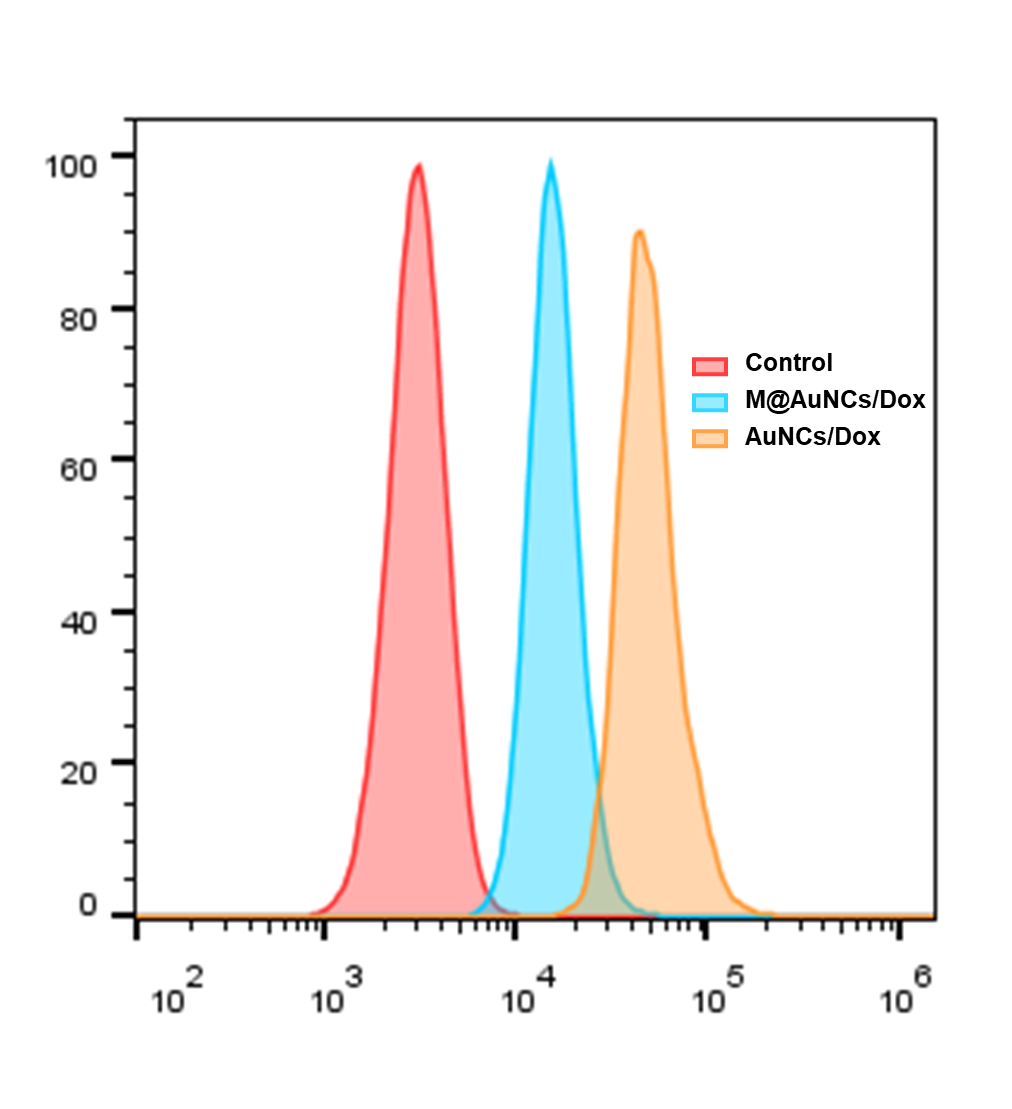


**Fig. S5.** Flow cytometry analysis of macrophage-like phenotype THP-1 cells after incubation with M@AuNCs/Dox or AuNCs/Dox for 4 h.


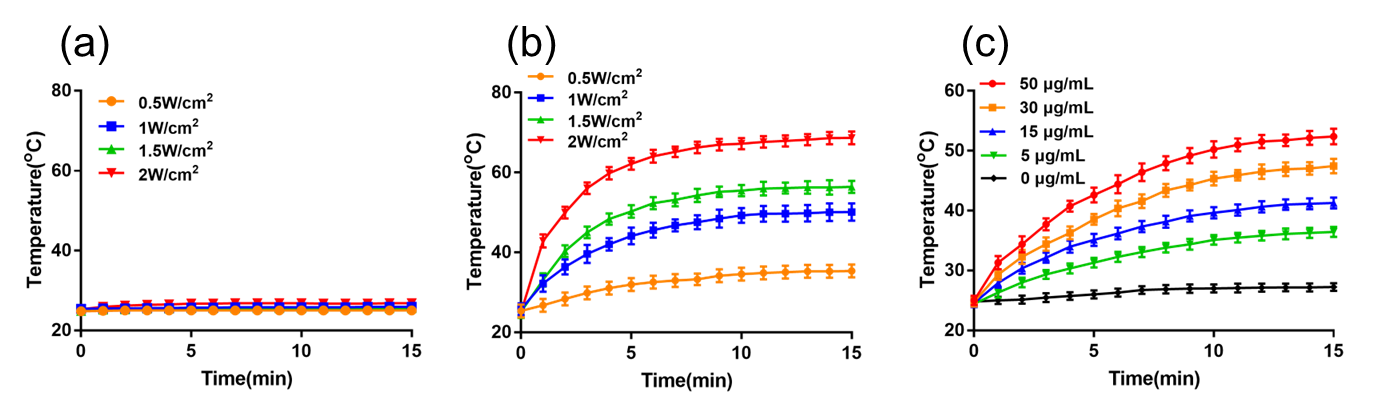


**Fig. S6.** Temperature elevation curve of **(a)** PBS and Col-M@AuNCs/Dox at **(b)** different NIR laser power irradiation and **(c)** different concentration.


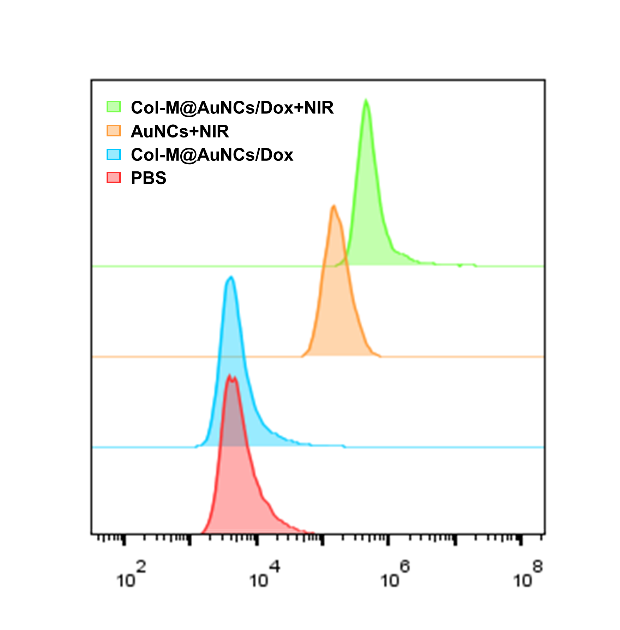


**Fig. S7.** Flow cytometry analysis of ROS generation in BxPC3 cells treated with Col-M@AuNCs/Dox, AuNCs+NIR, Col-M@AuNCs/Dox+NIR.


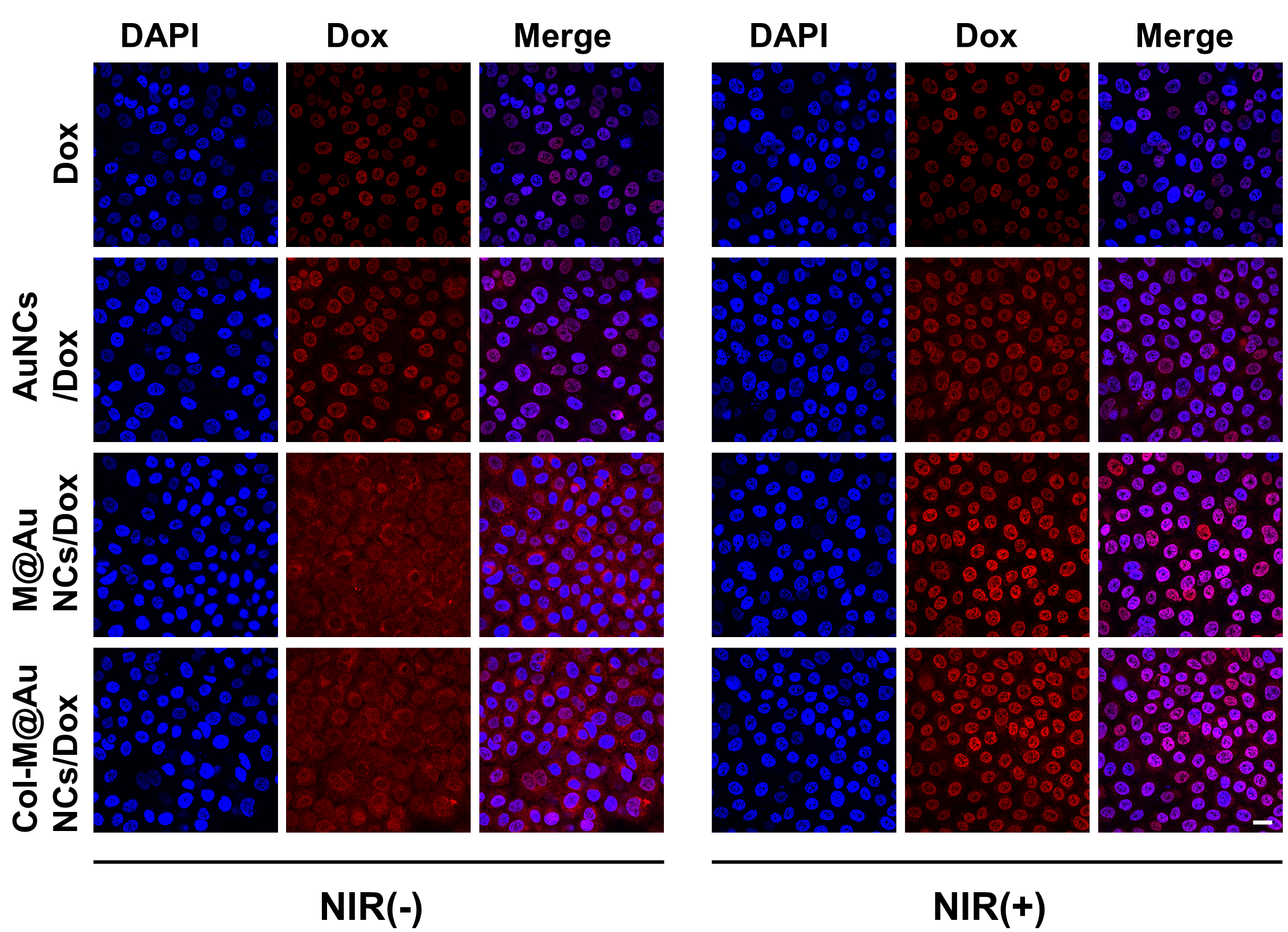


**Fig. S8.** CLSM observation of Dox release profiles of free Dox, AuNCs/Dox, M@AuNCs/Dox and Col-M@AuNCs/Dox in BxPC3 cells with or without NIR irradiation (Scale bar = 20 μm).


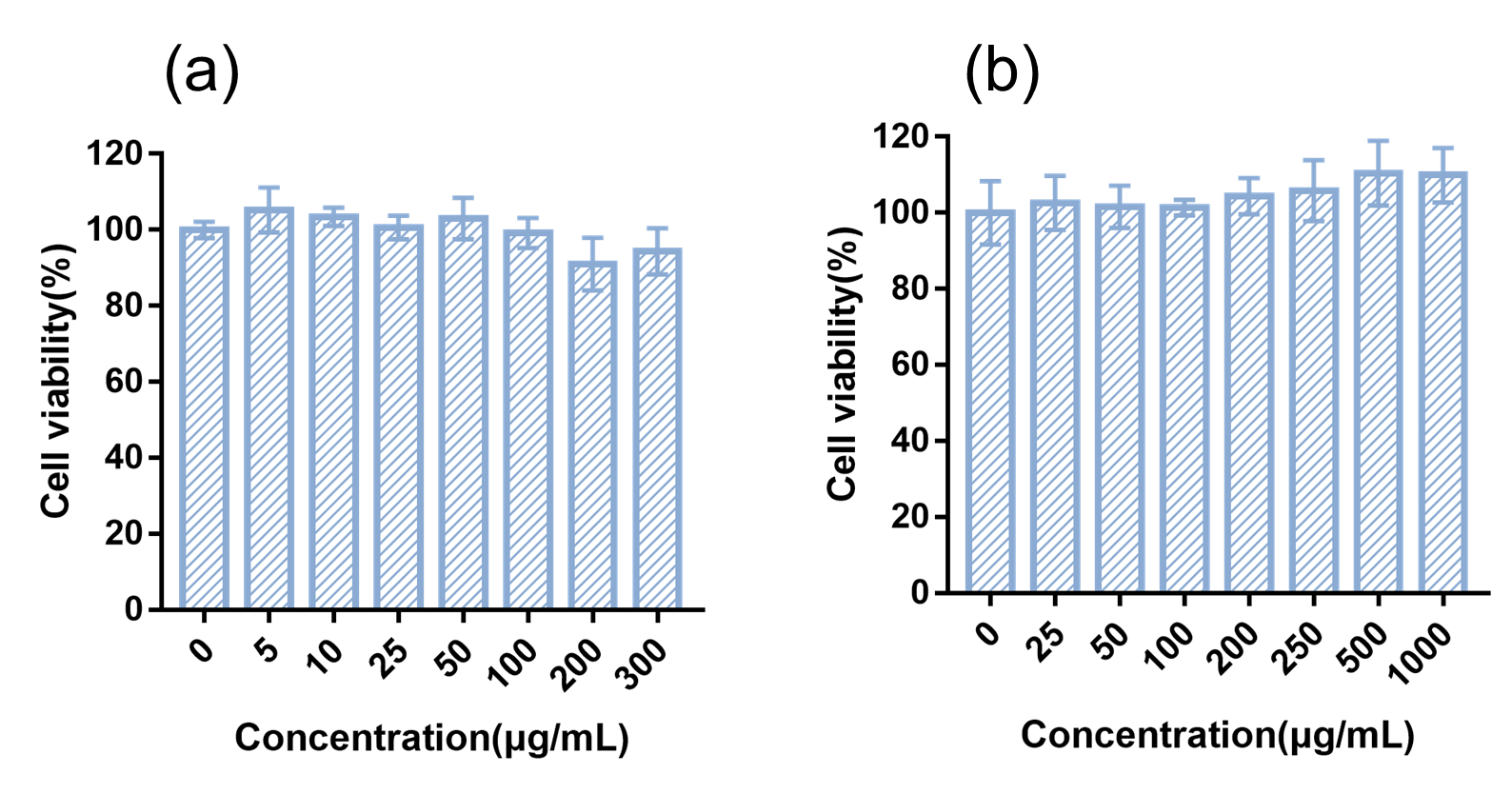


**Fig. S9.** Cell viability of BxPC3 cells treated with (**a**) AuNCs and (**b**) collagenase at different concentration.


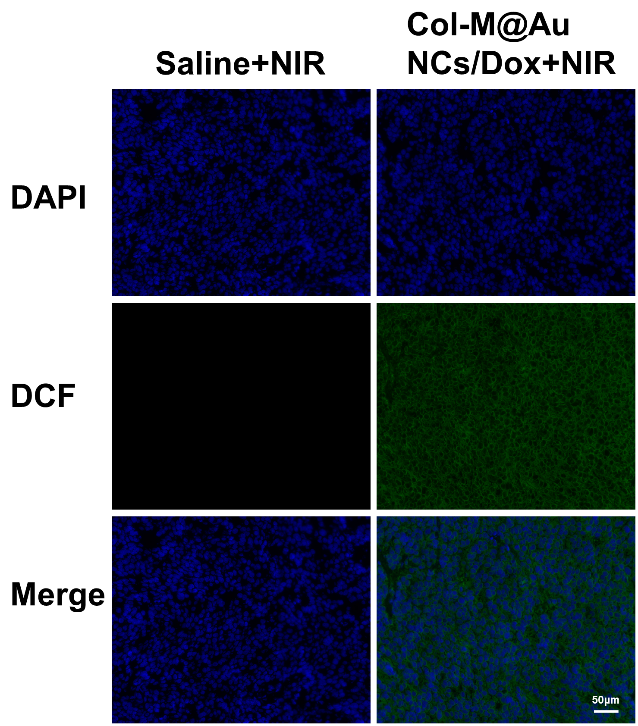


**Fig. S10.** ROS detection in BxPC3 tumor slices observed by CLSM.


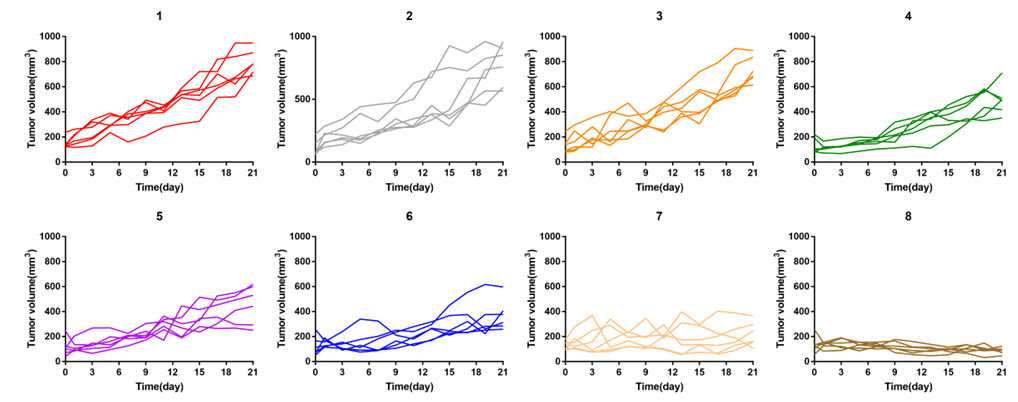


**Fig. S11.** Tumor spaghetti curves of BxPC3 tumor bearing mice with different treatment (n = 6). 1: Saline; 2: Saline+NIR; 3: AuNCs; 4: Dox; 5: AuNCs+NIR; 6: AuNCs/Dox+NIR; 7: M@AuNCs/Dox+NIR; 8: Col-M@AuNCs/Dox+NIR.


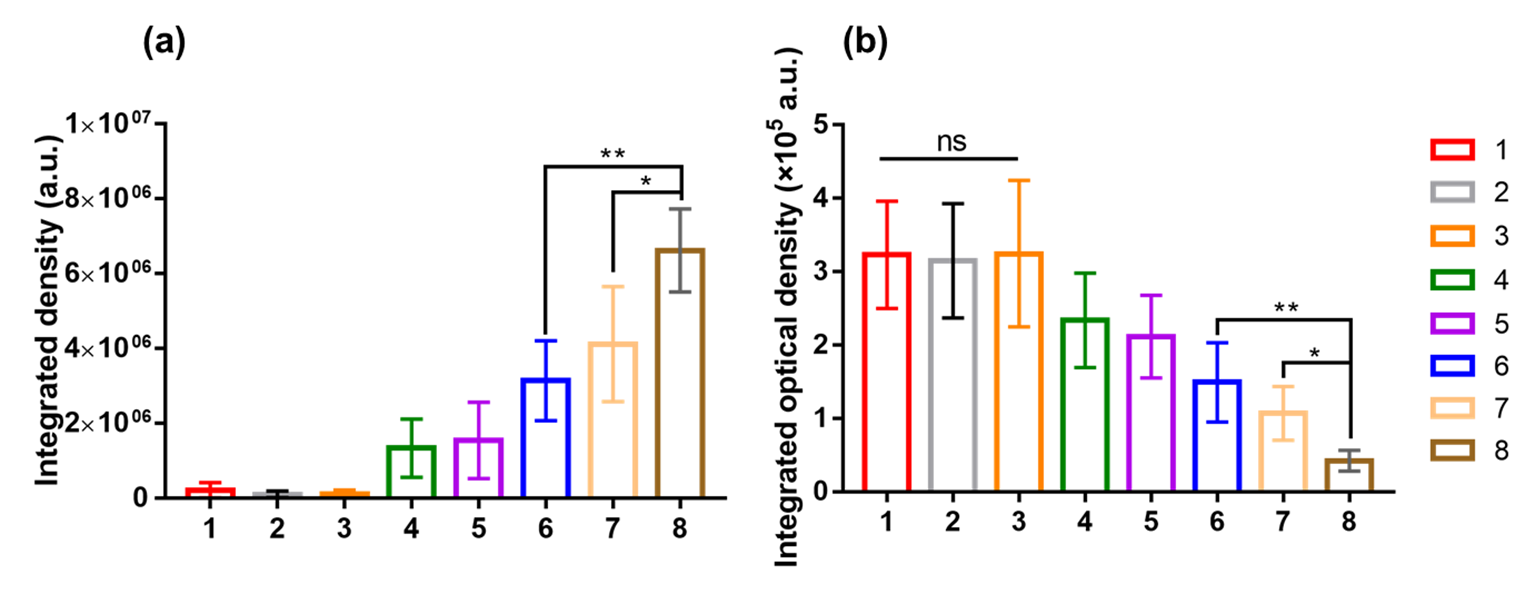


**Fig. S12.** Semi-quantitative analysis of **(a)** TUNEL and **(b)** Ki67 staining images. 1: Saline; 2: Saline+NIR; 3: AuNCs; 4: Dox; 5: AuNCs+NIR; 6: AuNCs/Dox+NIR; 7: M@AuNCs/Dox+NIR; 8: Col-M@AuNCs/Dox+NIR.


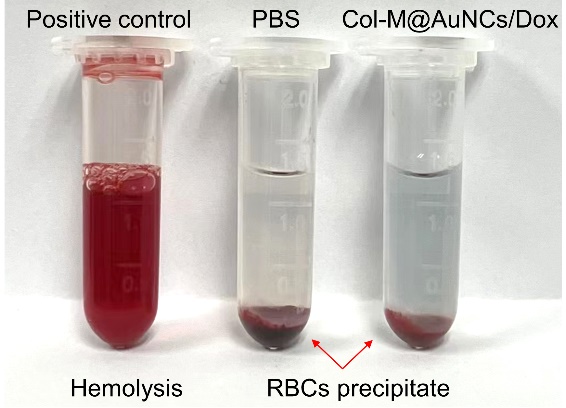


**Fig. S13.** Digital pictures of RBCs incubated with Triton X-100 (positive control), PBS (negative control) and Col-M@AuNCs/Dox after centrifugation.

**Table 1**

| **Hematological indexes** | **Saline** | **Col-M@AuNCs/Dox** |
| --- | --- | --- |
| RBC (10^12/L) | 8.14 ± 0.1552 | 8.41 ± 0.1582 |
| HGB (g/L) | 146.7 ± 2.906 | 145.7 ± 3.48 |
| MCV (fL) | 42 ± 0.3 | 41.73 ± 0.9387 |
| HCT (%) | 34.17 ± 0.5783 | 35.13 ± 1.417 |
| MCH (pg) | 18 ± 0.1155 | 17.4 ± 0.2 |
| MCHC (g/L) | 429.3 ± 1.333 | 415 ± 10.69 |
| WBC (10^9/L) | 7.37 ± 0.8381 | 6.77 ± 0.9642 |
| PLT (10^9/L) | 418 ± 88.75 | 436.3 ± 17.07 |

Hematological analysis of mice post-injection of Col-M@AuNCs/Dox and saline.

**
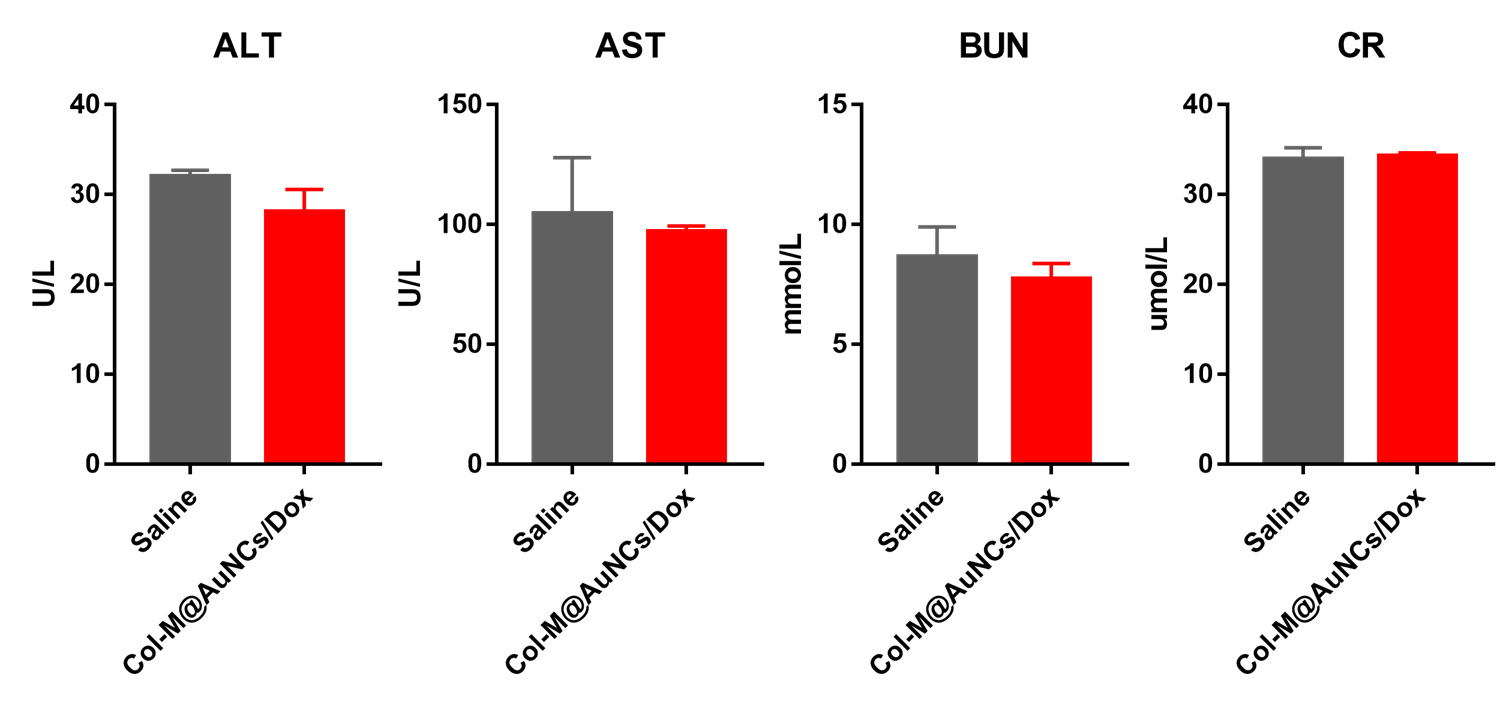
**

**Fig. S14.** Blood biochemical test of mice post-injection of Col-M@AuNCs/Dox and saline.


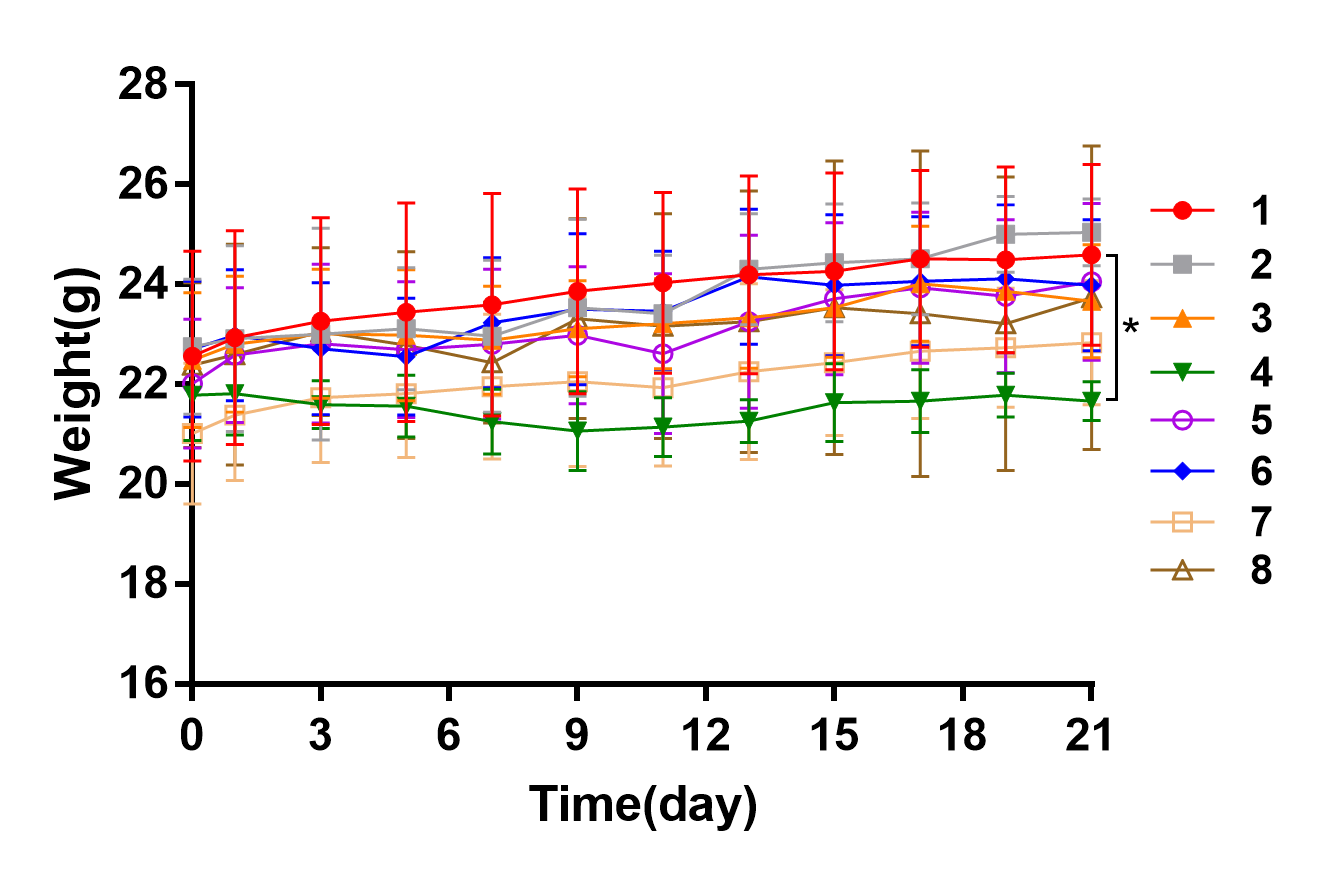


**Fig. S15.** Body weights of the mice during treatment (n = 6). 1: Saline; 2: Saline+NIR; 3: AuNCs; 4: Dox; 5: AuNCs+NIR; 6: AuNCs/Dox+NIR; 7: M@AuNCs/Dox+NIR; 8: Col-M@AuNCs/Dox+NIR.


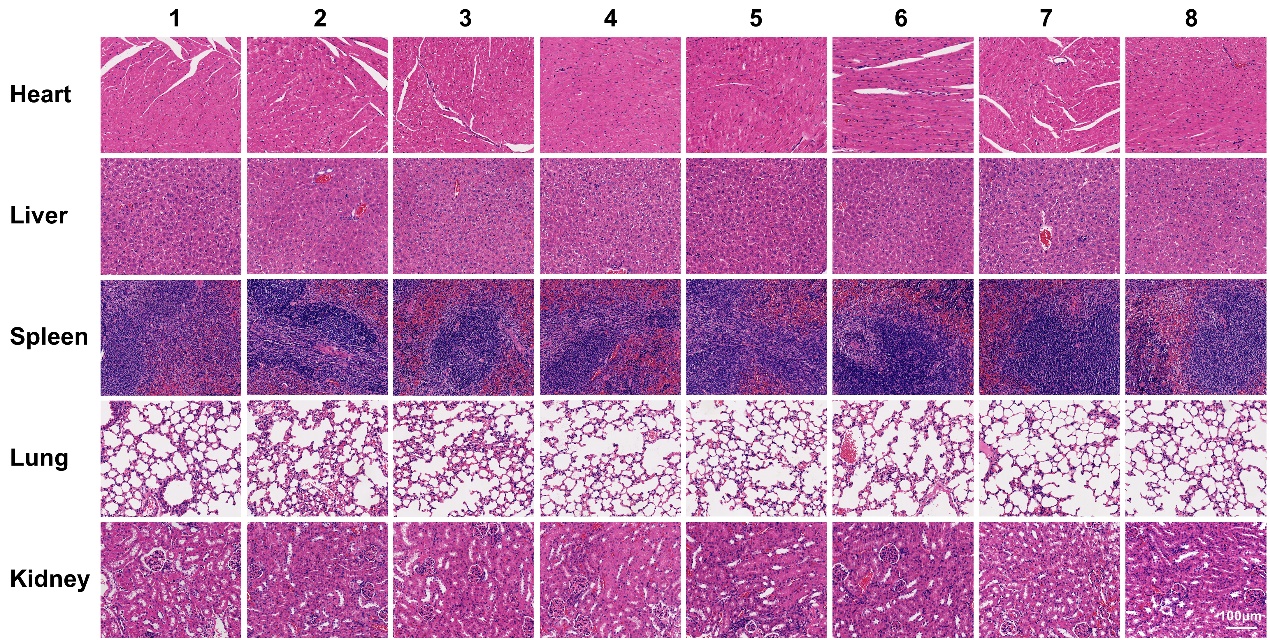


**Fig. S16.** H&E staining of the major organs (lung, liver, spleen, kidney, heart) extracted from BxPC3 tumor bearing mice after the 21 days treatment. 1: Saline; 2: Saline+NIR; 3: AuNCs; 4: Dox; 5: AuNCs+NIR; 6: AuNCs/Dox+NIR; 7: M@AuNCs/Dox+NIR; 8: Col-M@AuNCs/Dox+NIR.


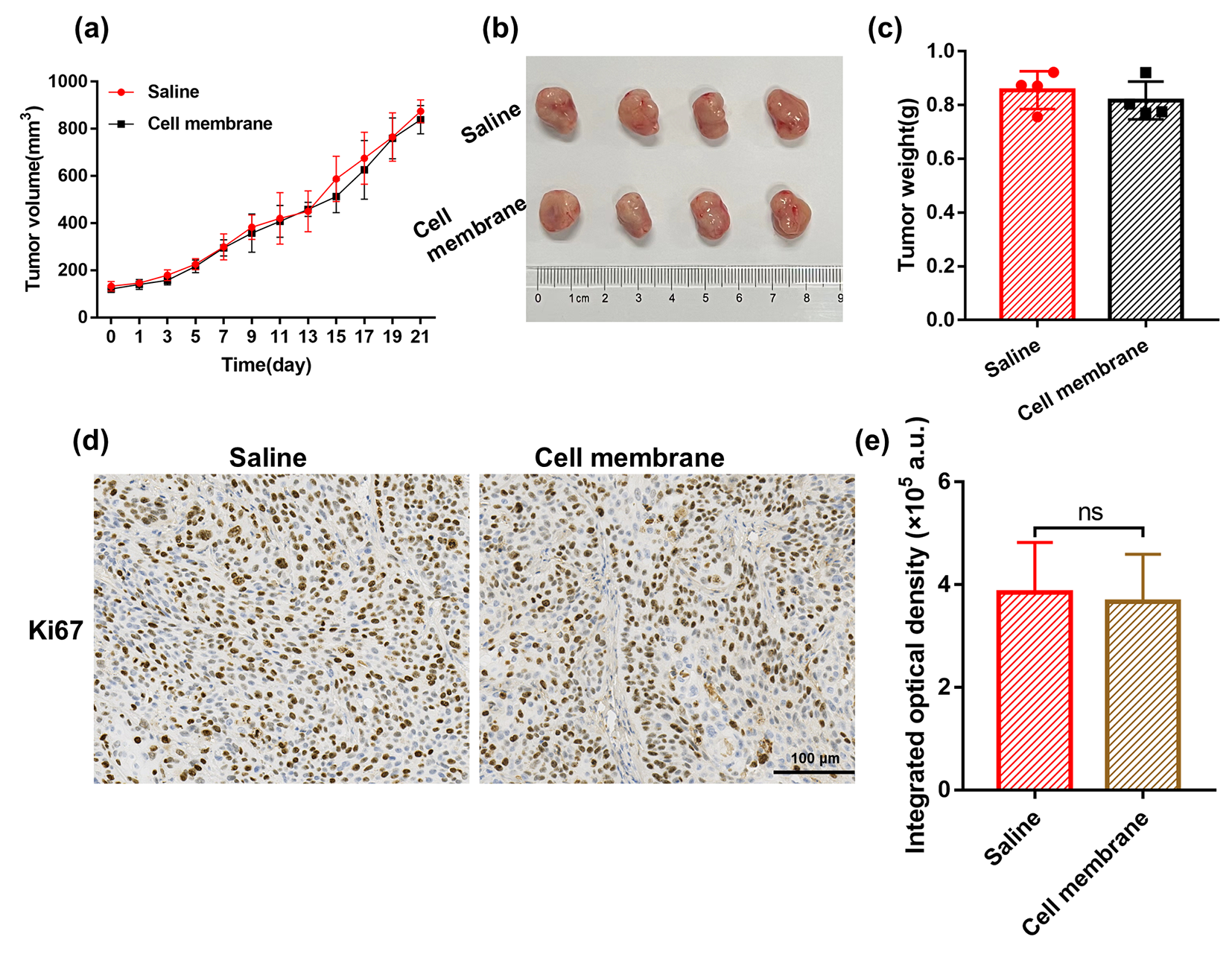


**Fig. S17.** **(a)** Tumor growth curves of mice after receiving saline and cell membrane treatment. **(b)** Images of the excised tumors on Day 21. **(c)** Weights of the excised tumors on Day 21. **(d)** Ki67 staining of tumors after mice receiving saline and cell membrane treatments. **(e)** Semi-quantitative analysis of the Ki67 staining images.

**
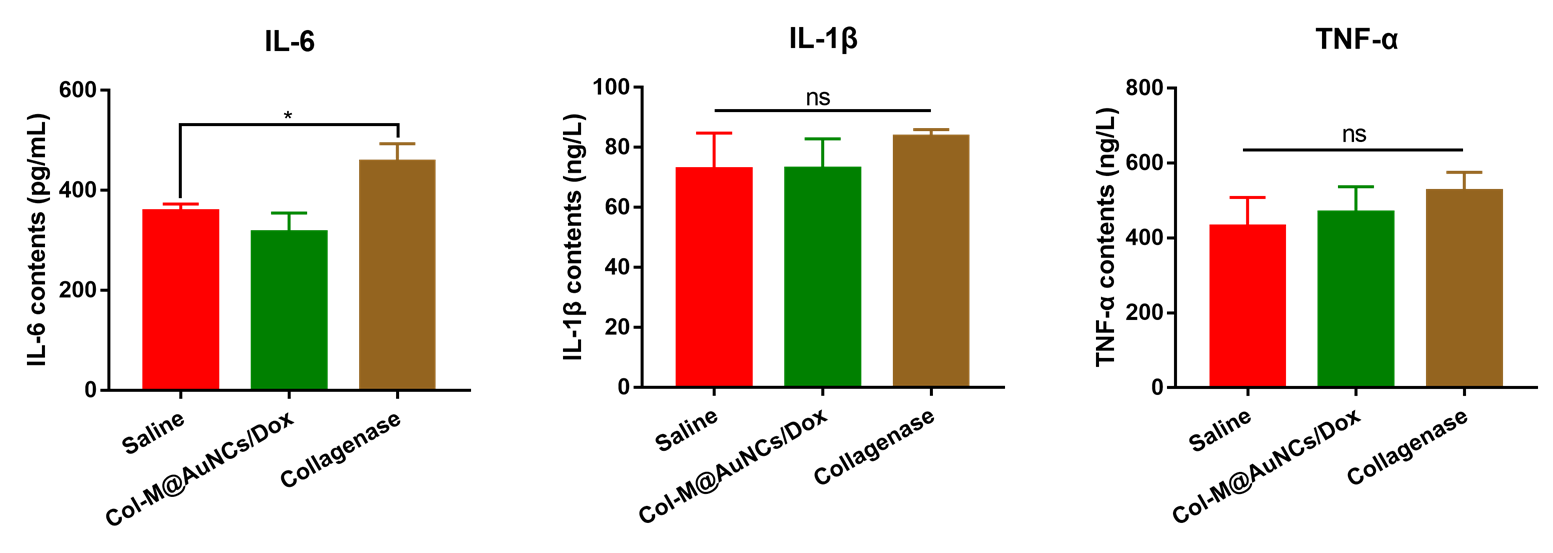
**

**Fig. S18.** IL-6, IL-1β and TNF-α contents in serum of mice post-injection of saline, Col-M@AuNCs/Dox and collagenase.

**
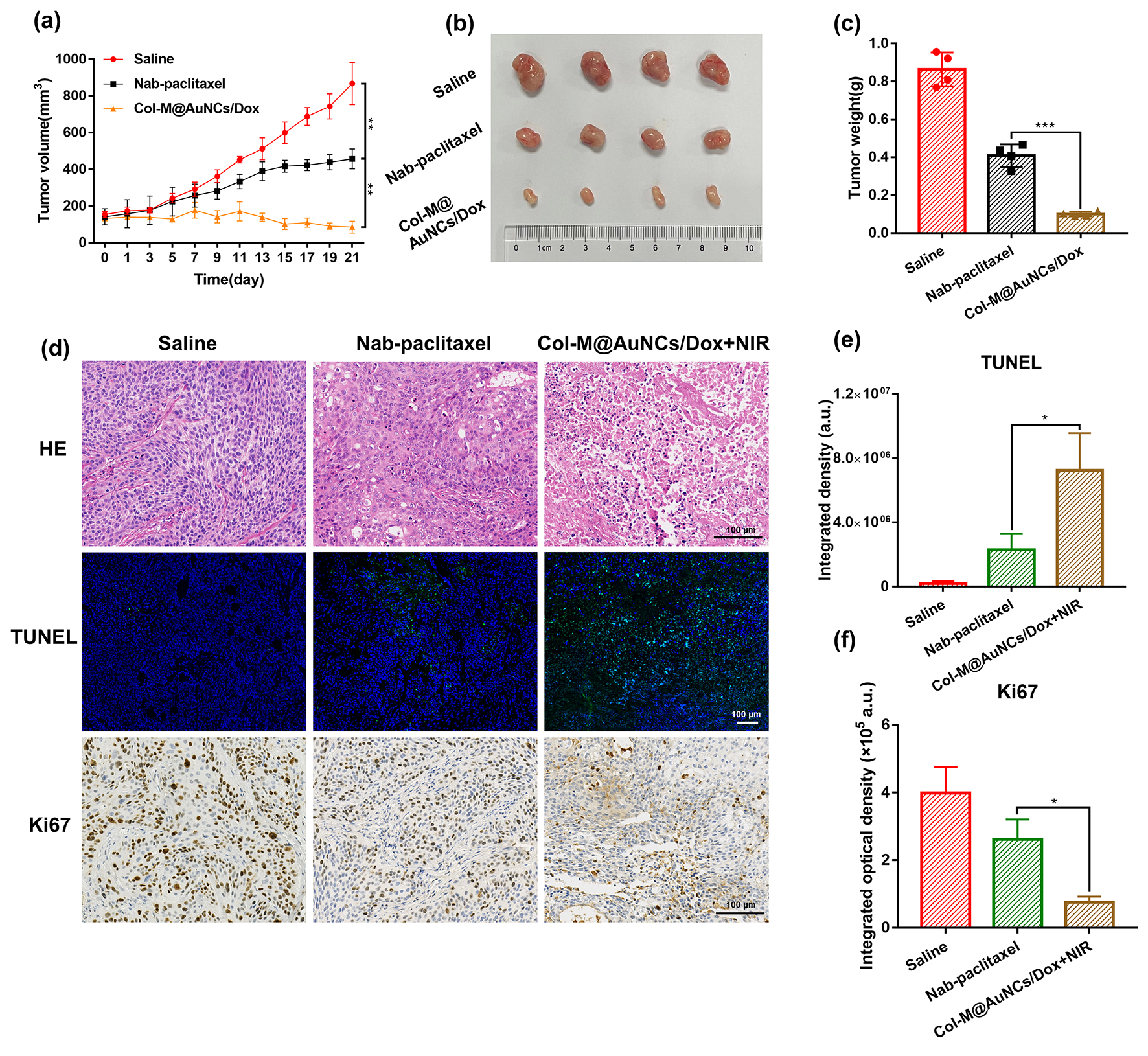
**

**Fig. S19. (a)** Tumor growth curves of mice after receiving saline, nab-paclitaxel and Col-M@AuNCs/Dox+NIR treatment. **(b)** Images of the excised tumors on Day 21. **(c)** Weights of the excised tumors on Day 21. **(d)** H&E, TUNEL and Ki67 staining of tumors after mice receiving different treatments. **(e)** Semi-quantitative analysis of TUNEL staining images. **(f)** Semi-quantitative analysis of Ki67 staining images.
